# PDKE-G: A Bio-Inspired Contributory Group Authenticated Key Exchange Grounded in Per-Member AlphaFold2 Protein Geometry

**DOI:** 10.3390/biomimetics11100753

**Published:** 2026-10-19

**Authors:** Abdullah Alabdulatif

**Affiliations:** Department of Computer Science, College of Computer, Qassim University, Buraydah 51452, Saudi Arabia; a.alabdulatif@qu.edu.sa

**Keywords:** authenticated group key exchange, bio-inspired cryptography, AlphaFold2, protein geometry, Burmester–Desmedt, elliptic-curve Diffie–Hellman, forward secrecy, ProVerif

## Abstract

Authenticated key exchange underpins secure communication, yet the structural complexity of biological macromolecules has rarely served as cryptographic material. The recently proposed Protein-Derived Key Exchange (PDKE) protocol binds AlphaFold2-predicted protein geometry to ephemeral elliptic-curve Diffie–Hellman for two parties. We introduce PDKE-G, a contributory group authenticated key exchange in which each member’s signing credential is derived from its protein geometry as a member-specific input: the long-term signing key is extracted with HKDF from the protein’s Cα–Cα distance matrix keyed by a per-member 128-bit secret seed, and a constant-round Burmester–Desmedt core wrapped by the Katz–Yung compiler with these protein-derived signatures yields a shared 256-bit session key with forward secrecy. We give a computational proof of security under Decisional Diffie–Hellman, KDF security of HKDF, pseudorandomness of the credential derivation, and strong unforgeability (SUF-CMA) of the signature, in the random-oracle model for the hash-to-curve and SHA-256 maps, and we verify the three- and four-party instantiations symbolically in ProVerif. Because every broadcast carries a per-member certified signature bound to the session context, PDKE-G prevents the honest-member insertion attack of the member-tampering setting within the scope of Corollary 1. An empirical study of 519 AlphaFold structures across seven organisms finds their canonical geometry descriptors all distinct, with a mean of 12.3 descriptive Shannon bits per residue pair. Because the selection space of known proteins is only about 2^27.7^, identity is anchored by the certified public key and the secret seed, which supplies the credential’s brute-force security. The protocol completes in a constant number of rounds independent of group size, with a logical-broadcast message count that is linear in the number of members; over point-to-point links both PDKE-G and pairwise composition are Θ(n^2^) in raw transmissions, so the advantages are the constant round count and the single contributory group key.

## 1. Introduction

Authenticated key exchange (AKE) lets parties with no prior shared secret agree on a fresh session key over a public network, and it underpins the confidentiality and integrity of modern communication [1]. While classical constructions draw their hardness from a small set of number-theoretic problems, the vast structural complexity of biological macromolecules has only recently been considered as a source of secret material. The PDKE protocol [2] took a first step in this direction, binding the three-dimensional geometry of a protein predicted by AlphaFold2 [3] to an ephemeral elliptic-curve Diffie–Hellman exchange, so that a high-entropy secret derived from a Cα–Cα distance matrix authenticates the session. That construction, however, is inherently two-party.

Many realistic settings are not two-party. A team of devices, a fleet of sensors, or a group of collaborators needs a single key shared by every member, established in one interactive protocol rather than a mesh of pairwise sessions. Composing a two-party protocol over all pairs costs a quadratic number of sessions and messages and still does not yield a single contributory group key. The natural question is therefore whether the bio-inspired approach of PDKE extends to the group setting while remaining efficient and provably secure.

We answer this question with PDKE-G, a contributory group authenticated key exchange in which the biological material is functionally integral to the identity derivation rather than illustrative. Instead of a single shared protein credential, each member derives its own long-term signing identity from the geometry of its own protein. The signing key is extracted, using HKDF, from the member’s Cα–Cα distance matrix keyed by a per-member secret seed, so that the protein geometry provides a distinct member-specific input while the certified public key and the secret seed anchor identity and provide brute-force security.

A constant-round Burmester–Desmedt core [4] provides the contributory key agreement, and the Katz–Yung compiler [5] lifts it to active security using these protein-derived signatures. Binding each broadcast to a session context of fresh nonces makes the agreement injective; within the scope of Corollary 1, this is what defeats the honest-member insertion attack shown against Burmester–Desmedt by Sun and Tian [6], though not every possible insider tampering.

The contributions of this paper are the following:

**Protocol:** We present PDKE-G, to our knowledge the first contributory group authenticated key exchange whose per-member signing credentials are derived from protein geometry as a member-specific input, achieving a shared 256-bit session key with forward secrecy in a constant number of rounds and a logical-broadcast message count linear in the group size; over point-to-point links, the raw transmission count remains *Θ*(*n*^2^).

**Security**: We give a game-based proof of security for general group size under Decisional Diffie–Hellman, KDF security of HKDF [7], pseudorandomness of the credential derivation, and strong unforgeability (SUF-CMA) of the signature, in the random-oracle model for the hash-to-curve and SHA-256 maps, separating key indistinguishability with forward secrecy from injective authentication under insider corruption. We also verify the three- and four-party instantiations symbolically in ProVerif [8], and we derive resistance to member-tampering insertion as Corollary 2.

**Empirical Grounding of the Credential:** Using 519 AlphaFold structures across seven organisms, we show that the unkeyed hashes of the 519 canonical geometry descriptors are all distinct and that the descriptors are structurally diverse, and we quantify the security-relevant selection entropy. Because that entropy is low for the space of known proteins, we make a per-member secret seed an explicit part of the design: the protein supplies a distinct member-specific input, while the certified public key and the secret seed anchor identity and provide the credential’s brute-force security.

**Binding Integrity:** We specify a certificate-level attestation that binds each member’s key to the canonical descriptor of its structure of record, so a relying party can recompute the descriptor and detect substitution or repointing of that structure. An executable control shows that this binding, unlike an ordinary public name, is at once reproducible under benign re-expression of the structure and sensitive to structural substitution (Section 4.2 and Section 5.6; Script S1 and Text S1).

**Reproducibility:** We provide a reference implementation, the ProVerif models, the entropy dataset, and the evaluation notebooks, all executable on a standard cloud runtime.

The remainder of the paper is organized as follows. Section 2 reviews related work. Section 3 fixes notation and building blocks. Section 4 specifies PDKE-G. Section 5 presents the security model, the symbolic verification, and the computational proof. Section 6 reports the implementation and evaluation. Section 7 discusses limitations and deployment, and Section 8 concludes.

## 2. Related Work

We situate PDKE-G against four strands of prior work: group key agreement and its authenticated variants, bio-inspired cryptography, the AlphaFold protein-structure resource, and the two-party PDKE protocol that this paper generalizes. We separate what is inherited from established results from what is new here.

Key agreement over a public channel originates with Diffie and Hellman [1]. Its extension to more than two parties was given by Burmester and Desmedt, whose conference key distribution system establishes a common contributory key in a constant number of broadcast rounds [4]; this construction remains the efficiency baseline for group key agreement and is the passively secure core that PDKE-G builds on. Turning such a passively secure protocol into one secure against an active network adversary is the role of generic compilers.

Katz and Yung give a scalable compiler that adds signatures and a session identifier to lift passive security to active security for an arbitrary number of parties, with forward secrecy [5], and Bresson, Chevassut, and Pointcheval analyze dynamic group Diffie–Hellman key exchange under standard assumptions with rigorous provable-security models [9]. Beyond the broadcast Burmester–Desmedt topology, tree-based group key agreement provides an alternative organization designed to support efficient membership changes, with rekeying work scaling logarithmically in the group size under the tree structure [10].

Insider security for group key exchange—the regime in which some participants are corrupted—was formally modelled by Katz and Shin [11], whose treatment informs our insider-security model. More recent work continues to sharpen these guarantees, for instance tightly secure group key exchange with perfect forward secrecy in both Diffie–Hellman and lattice settings [12]. PDKE-G follows this compiler pattern—a Burmester–Desmedt core authenticated with roster- and session-bound per-member signatures—and augments it with nonce precommitment and signed key confirmation; it contributes not a new group-key-agreement primitive but a specific, protein-grounded instantiation of the compiler’s per-member signing identities.

Within this line, Sun and Tian showed that the broadcast and cyclic Burmester–Desmedt protocols admit a member-tampering attack when two corrupted members belong to two groups, A and B: they can cause honest members of group B to believe that uninvolved members of group A participated and share the group key, and they proposed a countermeasure [6]. PDKE-G targets the corresponding honest-member insertion goal by assigning each participant a distinct PKI-certified signing key and binding every flow to the roster and session context; Corollary 2 precisely states the scope of the resulting resistance.

A separate line of work draws cryptographic constructions from biological systems. Within the protein- and proteinoid-based branch most relevant to PDKE-G, a recent example is the proteinoid-assembly approach of Mougkogiannis, Ghadafi, and Adamatzky, which investigates proteinoid assemblies as a substrate for cryptographic primitives [13]. Other biological substrates have likewise been explored, such as DNA-based cryptographic key generation [14]; these target different primitives and inputs than the structural-geometry identities used here. That direction differs from ours in both mechanism and goal: it does not aim at contributory group authenticated key exchange, and it does not use predicted three-dimensional molecular geometry as a per-member authentication identity.

PDKE-G instead treats AlphaFold-predicted Cα geometry as a member-specific local input to credential derivation, while the cryptographic strength is carried by standard primitives and a secret seed, as formalized in the credential construction and security assumptions below. To our knowledge, no prior bio-inspired construction provides the specific conjunction claimed here: a contributory group authenticated key exchange, with a computational proof for general group size, whose per-member signing credentials are derived from AlphaFold-predicted protein geometry as a member-specific input.

The data substrate underlying the credential is the accurate protein-structure prediction of AlphaFold2 [3] and the AlphaFold Protein Structure Database, whose 2024 release covers over 214 million sequences [15]; structure prediction has since extended to biomolecular complexes with AlphaFold 3 [16], though the present work uses only single-chain Cα geometry. This coverage is what makes the design explicit about its security basis: because the structures are public and enumerable, the selection space of known proteins is small (Section 6.2), so the geometry cannot serve as a secret. PDKE-G therefore protects the long-term signing credential through the secret per-member seed, anchors identity through the certified public key, and derives session-key confidentiality from the ephemeral group secret under the stated DDH and KDF assumptions; the database is used only as a source of distinct, reproducible, member-specific descriptors.

The immediate predecessor is the two-party PDKE protocol, which binds AlphaFold2-predicted protein geometry to an ephemeral elliptic-curve Diffie–Hellman exchange so that a protein-derived secret authenticates the session [2]. That construction is inherently two-party: composing it over all pairs of a group costs a quadratic number of sessions and messages and still yields only pairwise keys rather than a single contributory group key. PDKE-G fills this gap by moving the bio-inspired idea into the group setting with a constant-round contributory core, per-member protein-derived signing identities, and a computational proof for general group size, complemented by the fixed-size symbolic verification in ProVerif [8]. An unbounded-*n* symbolic analysis in Tamarin [17] and a post-quantum hybrid using a standardized lattice key-encapsulation mechanism [18] remain as future work.

The group-key-agreement and compiler machinery PDKE-G relies on is mature and is invoked as such; the novelty of this paper is the protein-grounded per-member instantiation, the conservative security scoping, and the empirical grounding of the credential.

## 3. Preliminaries

This section fixes the notation and the cryptographic building blocks used throughout. All primitives are pinned by the single suite identifier suite-ID of Section 4.1; the normative specifications of the protocol messages, the encoding, and the credential derivation are given in the protocol specification, and the security model with its assumptions in the security analysis. Here we recall only what those sections rely on, using the same symbols.

### 3.1. Notation and Cryptographic Setting

We write *λ* for the security parameter; PDKE-G targets a 128-bit level. A function is *negligible* if it is eventually smaller than every inverse polynomial in *λ*, and all adversaries are probabilistic polynomial-time (PPT). For a finite set *S*, $x\overset{R}{\leftarrow }S$ denotes uniform sampling of *x* from *S*; for a bit length *b*, {0,1}*^b^* denotes the set of *b*-bit strings, so an *L*-octet string lies in {0,1}^8^*^L^*. Concatenation is ∥. I2OSP(*x*,𝓁) is the 𝓁-octet big-endian encoding of a non-negative integer *x*, and OS2IP its inverse [19]; *u*32(*x*) = I2OSP(*x*,4). All structured values are serialized with the canonical, injective, length-prefixed encoding Encode(⋅) specified in Section 4.1, under which distinct field sequences never collide; elliptic-curve points use the 65-byte uncompressed SEC1 encoding [20].

The protocol operates over the NIST P-256 curve [21]. We write 𝔾 for its group of points of prime order *q*, *G* for the standard base point, 𝒪 for the point at infinity, and 𝔾_nz_ = 𝔾\{𝒪} for the non-identity elements. Scalars live in ℤ*_q_*, and $Z_{q}^{*}$ denotes the non-zero scalars. P-256 has cofactor one, so every non-identity element generates 𝔾. Ephemeral elliptic-curve Diffie–Hellman uses fresh scalars $a\overset{R}{\leftarrow }Z_{q}^{*}$ and the point *aG* over the standard base point in the generic exposition, or aG^*^ over the per-session base point in PDKE-G.

Using the additive notation of the curve, the *Decisional Diffie–Hellman (DDH)* assumption in 𝔾 states that no PPT distinguisher separates (*g*,*xg*,*yg*,(*xy*)*g*) from (*g*,*xg*,*yg*,*zg*) for a generator *g* and $x,y,z\overset{R}{\leftarrow }Z_{q}$ except with advantage ${\mathrm{Adv}}_{G}^{\mathrm{ddh}}$ negligible in *λ*; this is formalized as Assumption 1 and the sole number-theoretic hardness the key-secrecy proof rests on.

The per-session base point is produced by the RFC 9380 SSWU hash-to-curve suite for P-256 (the random-oracle suite P256_XMD:SHA-256_SSWU_RO_, as opposed to the non-uniform suite) [22], written hash_to_curve(⋅) and instantiated with the protocol’s domain-separation tag. The map is deterministic, uses no trial-and-error rejection, and returns a valid curve point; the implementation validates the returned point and aborts on the point at infinity. In the computational proof this map is modelled as a random oracle into 𝔾_nz_ (equivalently, onto the generators of the prime-order group 𝔾), as stated in Assumption 4.

### 3.2. Authentication, Hashing, and Key Derivation

SHA-256 [23] is the hash used to form the roster and session identifiers and the transcript hash; in the proof it is modelled as a random oracle (Assumption 6), which notably offers collision resistance. HMAC-SHA-256 is the associated MAC and, keyed by a secret input, a pseudorandom function. Key derivation uses HKDF [7,24] in its two stages: HKDF-Extract(salt,IKM) = HMAC-SHA-256(salt,IKM) produces a pseudorandom key, and HKDF-Expand(PRK,info,*L*) is a variable-length PRF; distinct info labels yield outputs that are jointly indistinguishable from independent random keys (the multi-output KDF security of Assumption 3). The credential extraction and the session-key separation both use these two stages exactly as fixed in Section 4.2 and Section 4.4.

Authentication uses ECDSA over P-256 [25], with signing and verification written Sig and Vf. The instantiation is pinned: deterministic per-signature nonces (RFC 6979 [26]), strict public-key and point validation on every received key, and a canonical low- *S*, fixed-length encoding I2OSP(*r*,32) ∥ I2OSP(*s*,32) that removes encoding ambiguity and rejects the direct high-*S* counterpart (*r*,*n* − *s*); it does not by itself establish strong unforgeability. Because the Katz–Yung compiler requires strong unforgeability, the security assumption is SUF-CMA of this instantiated scheme (Assumption 2). The concrete theorem is accordingly conditional on this explicit assumption; RFC 6979 and low-S verification do not by themselves establish it, and a signature scheme with a published SUF-CMA bound may be used instead.

Bulk group data after key agreement is protected with AES-256-GCM [27] under the derived application key *K*_app_. AES-GCM is an authenticated encryption with associated data (AEAD) scheme whose security requires per-key nonce uniqueness across all senders; the per-sender nonce construction and the mandatory key-renewal rule that enforce this are specified in Section 4.4.

### 3.3. Group Key Agreement and Protein-Derived Credentials

The contributory core is the constant-round Burmester–Desmedt (BD) group key-agreement protocol [4], in which each member broadcasts an ephemeral contribution and a ring value and every member reconstructs a common group element; its passive security under DDH is a published result invoked as a black box in Section 5.4. Active security is obtained with the Katz–Yung (KY) compiler [5], which lifts a protocol secure against a passive adversary to one secure against an active network adversary by signing every flow and binding it to a session identifier. PDKE-G instantiates the compiler’s signatures with the per-member protein-derived identities defined below.

Each member’s long-term credential is derived from the three-dimensional geometry of a protein whose model is retrieved from the AlphaFold Protein Structure Database [3,15]. From the model’s Cα atoms a quantized upper-triangular Cα–Cα distance descriptor *D_i_* is formed under the normative canonicalization profile PDKE-G-Dmatrix-v1 (Section 4.2). The descriptor is a member-specific local input, a public content-derived commitment to the member’s structure of record whose binding is made publicly verifiable by the certificate attestation of Section 4.2; the long-term signing scalar is derived as *k*_bio,_*_i_* = HKDF-Extract(salt = *r_i_*,IKM = *D_i_*) with a secret 128-bit seed *r_i_* in the HMAC key position, followed by rejection-sampled hash-to-scalar to *d_i_* ∈ [1,*q* − 1] and *Q_i_* = *d_i_G* certified in the PKI. The security consequence of keying the extraction with the secret seed is formalized as Assumption 5, and the descriptor is characterized empirically later.

## 4. The PDKE-G Protocol

### 4.1. Overview and Design Rationale

**Figure 1:**
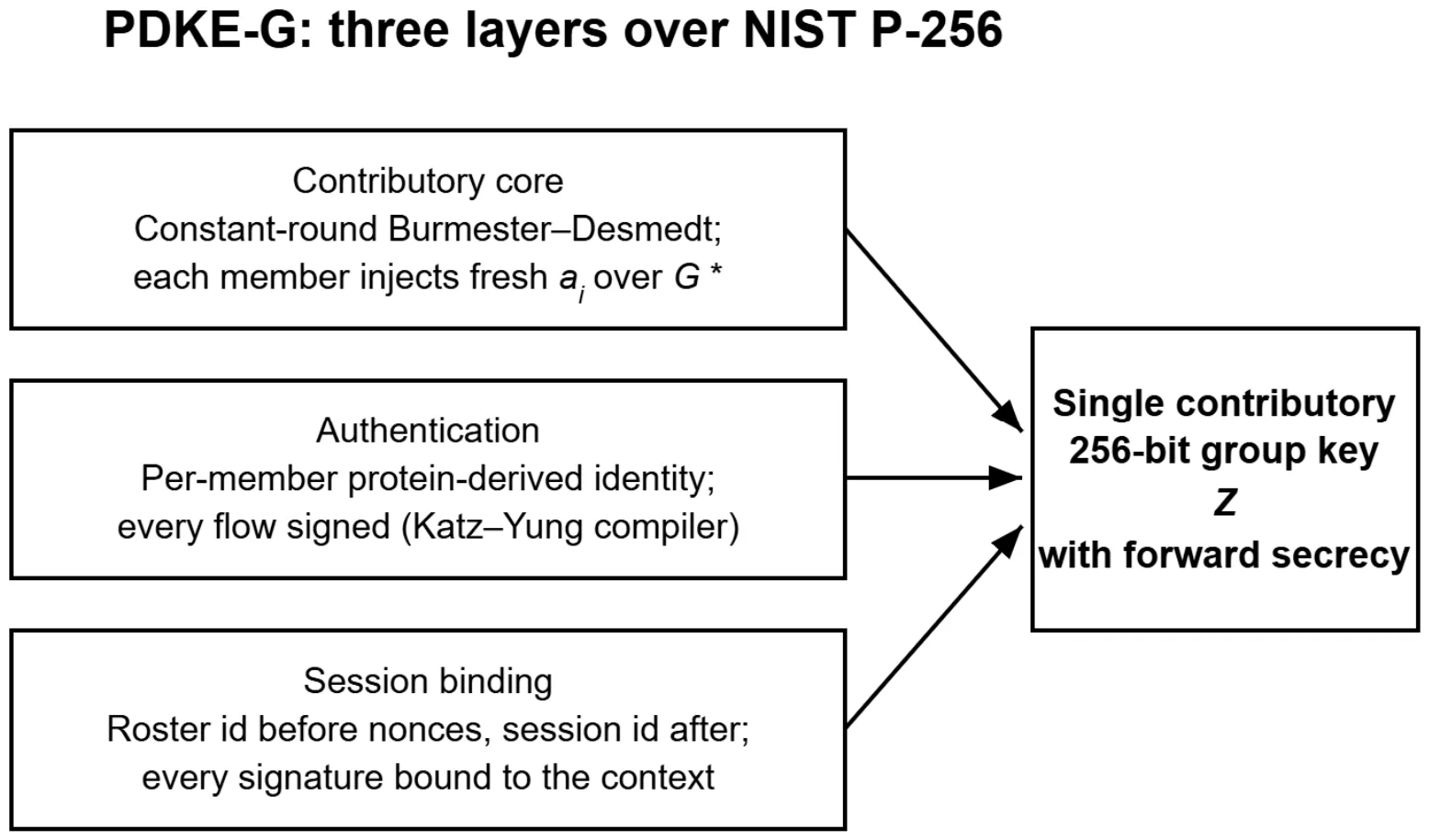
PDKE-G combines three layers over NIST P-256: a constant-round Burmester–Desmedt contributory core, per-member protein-derived signing identities under the Katz–Yung compiler, and roster- and session-binding, yielding a single 256-bit group key with forward secrecy.

PDKE-G establishes a single contributory session key among *n* ≥ 3 members. Its design combines three layers (Figure 1). The first layer is a contributory key-agreement core: the constant-round Burmester–Desmedt (BD) protocol [4], in which every member injects fresh ephemeral randomness. Under honest execution the resulting key depends on every member’s contribution; the stronger insider guarantees are stated and proved in the security analysis rather than assumed here. The second layer is authentication: rather than a single shared credential, every member holds a long-term signing identity derived from the geometry of its own protein, and every key-exchange contribution (Rounds 0 to 2) is digitally signed, following the Katz–Yung compiler [5], which turns a protocol secure against a passive adversary into one secure against an active network adversary.

The Katz–Yung compiler justifies active-network authentication for uncorrupted participants; the stronger malicious-insider, member-tampering, and injective-authentication properties claimed for PDKE-G rest on the separate analysis of Section 5. The final confirmation message combines a session-key-derived HMAC with an ECDSA signature, providing both key-possession evidence and sender attribution. The third layer is session binding: the members fix a roster identifier before the nonce round and a full session identifier after it. The Round 0 signatures are bound to the roster identifier, and the Round 1 and Round 2 signatures to the session identifier.

This binding is intended to prevent replay and cross-session reinterpretation of messages; that it yields injective authentication and defeats the member-tampering insider attack [6] is established by Theorem 2 and Corollary 2.

Throughout, the group operates over the NIST P-256 curve [21]; 𝔾 denotes its group of points of prime order *q*, and *G* its standard base point. A fixed suite identifier suite − ID pins the curve, the RFC 9380 map with its domain-separation tag, the signature scheme, the MAC, the key-derivation function, and the transport cypher. All structured values are serialized with a single canonical, injective encoding Encode(⋅) in which every field is prefixed by its length as a 4-byte big-endian integer: integer fields such as *n*, the index *i*, and counters are fixed 8-byte big-endian; curve points use the 65-byte uncompressed SEC1 encoding; and byte strings and ASCII labels are carried verbatim.

A tuple is the length-prefixed concatenation of its fields, so distinct field sequences never collide. ECDSA signatures use the fixed-length encoding I2OSP(*r*,32) ∥ I2OSP(*s*,32) in canonical low-*S* form. Members agree on a canonical roster order, namely ascending order of the encoded public keys Encode(*Q_i_*), so that every party forms identical identifiers and the same ring. For reference throughout, 𝒪 denotes the point at infinity and 𝔾_nz_ the set of non-identity elements of 𝔾 (every point except 𝒪), the group size satisfies 3 ≤ *n* ≤ 2^32^ − 1 with member indices 1 ≤ *i* ≤ *n*, all confirmation HMAC tags are the full 32-byte output of HMAC-SHA-256, and the AES-GCM authentication tag length is fixed by the suite at 128 bits.

The lower bound *n* ≥ 3 is not merely conventional: at *n* = 2 the cyclic neighbours *i* + 1 and *i* − 1 coincide modulo *n*, so *z_i_*_+1_ − *z_i_*_−1_ = 𝒪 and every ring value *X_i_* = 𝒪, which the acceptance rules of Section 4.4 reject; the protocol is therefore defined only for *n* ≥ 3, consistent with the experimental range *n* = 3, …, 50.

### 4.2. Setup: Per-Member Protein-Derived Identity

Each member *U_i_* is associated with a protein whose AlphaFold2 model is retrieved from the AlphaFold Protein Structure Database [3,15]. Because the long-term credential depends on the exact bytes of *D_i_*, the descriptor is fixed by the normative canonicalization profile PDKE-G-Dmatrix-v1 below, which two independent implementations can follow to reproduce identical bytes. The profile itself is versioned, and a deployment MAY instead cite a frozen profile by its SHA-256 checksum.

1. *Source, Format, and Integrity*: The input is the AlphaFold Protein Structure Database predicted model for the member’s UniProt accession in mmCIF format (the database’s canonical format), with the database release and model version recorded explicitly and the downloaded model file pinned by its SHA-256 digest. Only ATOM records for the twenty standard amino-acid residues are considered; HETATM records and hydrogen atoms are ignored.
2. *Chain, Model, and Alternate Locations:* The first model is used; a single chain is selected, by default the label_asym_id of the first standard-residue Cα encountered in _atom_site loop order (so the choice does not depend on library-specific chain ordering), and its identifier is recorded. Where a Cα atom carries alternate-location indicators, the conformer of highest occupancy is taken, with ties broken by the lexicographically smallest alternate-location identifier.
3. *Residue Ordering and Missing Data*: Residues are ordered by the pair (author residue sequence number ascending, then insertion code in ASCII order); exactly one Cα atom is taken per residue. Residues that lack a resolved Cα are excluded, and the retained Cα atoms, in that order, define the index set 1,…,*m*. Alternate-location conformers of a single residue are not two residues: they are resolved to one Cα by the occupancy rule of step 2. If, after that resolution, two genuinely distinct residues still share the same sequence number and insertion code—detected as more than one non-alternate Cα for one key, or a single key carrying more than one label_comp_id or label_seq_id—the profile aborts rather than guess an order.
4. *Coordinates and Distances:* Each Cα coordinate is read from the mmCIF Cartn_x/y/z fields, which are expressed to 0.001 Å, and is parsed as an exact integer number of milliångström, round-half-to-even (1000 ⋅ value). For 1 ≤ *a* < *b* ≤ *m*, the squared distance is then computed exactly in integer arithmetic as *s_ab_* = (*Δx*)^2^ + (*Δy*)^2^ + (*Δz*)^2^, in units of 10^−6^ Å^2^, with no floating-point rounding.
5. *Quantization:* Each distance is quantized to 0.01 Å as $q_{\mathrm{ab}}=round\text{-}half\text{-}to\text{-}even\left(\sqrt{s_{\mathrm{ab}}}/10\right)$: choose the integer bin *q_ab_* whose rounding interval contains *s_ab_*: for *q_ab_* ≥ 1 the closed interval ${\left(10q_{\mathrm{ab}}-5\right)}^{2}\leq s_{\mathrm{ab}}\leq {\left(10q_{\mathrm{ab}}+5\right)}^{2}$, and 0 ≤ *s_ab_* ≤ 25 for the boundary case *q_ab_* = 0 (where the lower bound is omitted). At a shared boundary $s_{\mathrm{ab}}={\left(10q_{\mathrm{ab}}\pm 5\right)}^{2}$ the two adjacent bins are both candidates, and the even bin is selected (round-half-to-even), which resolves the choice uniquely. Because *s_ab_* is an exact integer, this determination is exact and independent of any square-root implementation, so no floating-point tie can arise near a half-unit boundary.
6. *Missing and Malformed Fields:* An alternate-location or occupancy field recorded as . or ? is treated as a single unlabelled conformer of occupancy 1; any non-numeric or absent Cartn_x/y/z value for a retained Cα causes the profile to abort rather than substitute a default.
7. *Serialization:* Write *u*32(*x*) = I2OSP(*x*,4) for the 4-octet unsigned big-endian encoding of a non-negative integer. The matrix order *m* and every quantized entry *q_ab_* are represented by *u*32(⋅), the 4-octet width covering all physically attainable Cα–Cα distances. Each *u*32(⋅) value is carried by Encode as a fixed 4-byte byte string, not as its general 8-octet integer field, so the profile’s width is authoritative for these entries. The entries are emitted in upper-triangular row-major order, that is, over the pairs (*a*,*b*) with 1 ≤ *a* < *b* ≤ *m* in lexicographic order.

The canonical descriptor is then given by Equation (1):

(1) *D_i_* = 𝖤𝗇𝖼𝗈𝖽𝖾(“PDKE-G-Dmatrix-v1”,*u*32(*m*),*u*32(*q*_12_),*u*32(*q*_13_),…,*u*32(*q*_(_*_m_*_−1) _*_m_*)).

**Figure 2:**
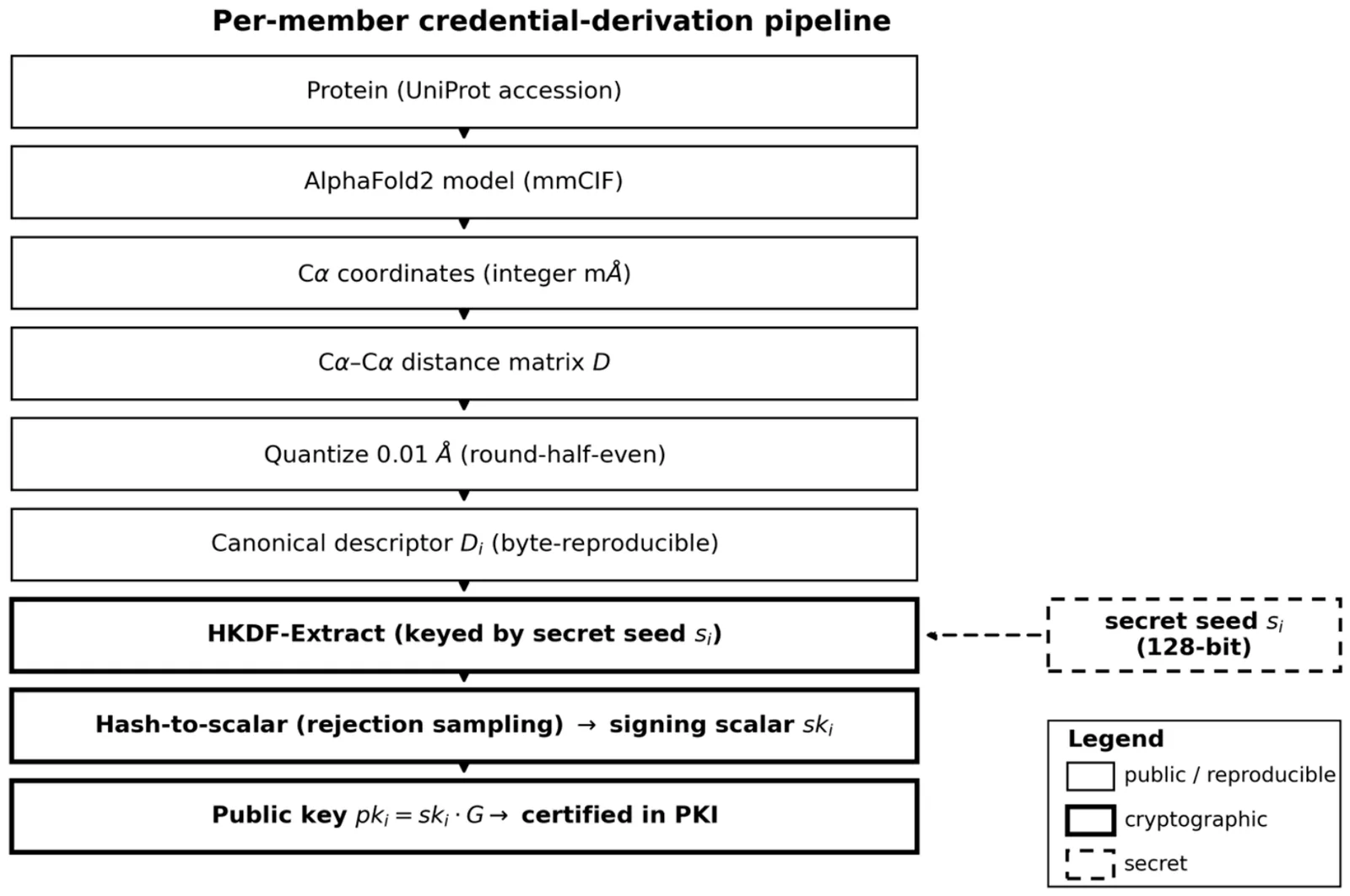
Per-member credential-derivation pipeline: the public, byte-reproducible chain from the protein to the canonical descriptor feeds an HKDF extraction keyed by the secret 128-bit seed, from which the signing scalar, the certified public key, and the PKI credential follow.

Every step above is integer-deterministic, so two independent implementations following this profile produce byte-identical *D_i_*; short descriptor test vectors are provided in the reproducibility package to check conformance. The member draws a secret, uniformly random seed $r_{i}\overset{R}{\leftarrow }\{0,1{\}}^{128}$, stored securely, and computes its long-term credential by HKDF extraction, Equation (2) [7,24]; the full derivation pipeline is shown in Figure 2. Here and below, $x\overset{R}{\leftarrow }S$ denotes uniform random sampling of *x* from the set *S*. The extraction is

(2) *k*_bio,_*_i_* = 𝖧𝖪𝖣𝖥-𝖤𝗑𝗍𝗋𝖺𝖼𝗍(salt = *r_i_*, IKM = *D_i_*) = 𝖧𝖬𝖠𝖢-𝖲𝖧𝖠-256(*r_i_*,*D_i_*).

Note that the secret seed occupies the HMAC key position of the extraction, so the derivation is a PRF evaluation keyed by *r_i_* rather than a reliance on the entropy of *D_i_*; the security consequence is formalized as Assumption 5. The design separates two roles. The protein geometry is a member-specific local input to credential derivation; it is not, by itself, a publicly verifiable biometric binding, since a verifier cannot check from *Q_i_* that any particular protein was used.

The 128-bit secret seed *r_i_* supplies the credential’s brute-force security. Because the selection space of known proteins is small (Section 6), a public salt would add no secrecy, so resistance to offline recovery of the long-term credential rests on the secret seed, not on the protein’s unpredictability. This is substantiated by the ablation study (Section 5.6): recovering the signing key costs 2^128^ regardless of whether the input is the real descriptor, a public accession string, or a constant, while a variant without the secret seed is recovered within the public selection space. Empirically, no collisions were observed among the 519 evaluated geometry encodings; this is an observation on the sample, not a universal collision-freedom guarantee.

From *k*_bio,_*_i_* the member derives its P-256 signing scalar by a fully specified hash-to-scalar with rejection sampling. Let *c* be an unsigned 64-bit counter, 0 ≤ *c* < 2^64^, encoded by Encode as the general 8-octet integer field (aborting if the counter space is exhausted). For *c* = 0,1,… set

(3) *T_c_* = 𝖧𝖪𝖣𝖥-𝖤𝗑𝗉𝖺𝗇𝖽(*k*_bio,_*_i_*, 𝖤𝗇𝖼𝗈𝖽𝖾(“PDKE-G ECDSA scalar”,suite-ID,*c*), 32),  *x_c_* = 𝖮𝖲2𝖨𝖯(*T_c_*),

with *T_c_* read as a 32-byte big-endian integer; the first *x_c_* with 1 ≤ *x_c_* < *q* is taken as *d_i_*, and *Q_i_* = *d_i_* *G*. Rejection sampling is used rather than modular reduction because reducing a uniform 256-bit value modulo *q* would bias the scalar: for P-256, 2^256^ − *q* is of order 2^224^, so the statistical distance is about 2^−32^, which is not negligible for a new specification; the rejection probability is likewise about 2^−32^, so a candidate is accepted on the first counter except with negligible probability.

The PKI binds the resulting public key *Q_i_* to the registered participant; each session roster contains exactly *n* distinct public keys bound by the PKI to the intended participants. A certificate-level attestation, specified here, makes this binding publicly checkable. At enrollment the certifier recomputes the canonical descriptor D_i = canon(structure) from the public structure referenced by the member, and signs, together with the member’s public key, the pair consisting of that structure reference and SHA-256(D_i). A relying party recomputes canon of the referenced public structure and compares the hash, so any substitution or repointing of the structure behind the reference invalidates the certificate (Section 5.6). The attestation binds the key to a specific, publicly reproducible structure of record; it does not certify the biological authenticity of the protein. Signatures use ECDSA over P-256 [25] with deterministic per-signature nonces (RFC 6979 [26]), strict public-key validation on every received key, and a canonical low-*S* signature encoding that removes encoding ambiguity and rejects the direct (*r*,*n* − *s*) high-*S* variant (it does not by itself establish strong unforgeability; see Assumption 2); signing and verification are written as Sig and Vf.

### 4.3. Session Identifier and Per-Session Base Point

Before the nonce round, the members form a roster identifier that binds the protocol version, the suite, the group size, and the entire roster of public keys,

(4) 𝗋𝗈𝗌𝗍𝖾𝗋𝖨𝖣 = 𝖲𝖧𝖠-256(𝖤𝗇𝖼𝗈𝖽𝖾(“PDKE-G-roster-v1”,suite-ID,*n*,*Q*_1_,…,*Q_n_*)),

and the Round 0 signatures are bound to rosterID. After the nonce round, the members extend this into a full session identifier that additionally binds every nonce,

(5) 𝗌𝗂𝖽 = 𝖲𝖧𝖠-256(𝖤𝗇𝖼𝗈𝖽𝖾(“PDKE-G-sid-v1”,suite-ID,*n*,*Q*_1_,…,*Q_n_*,*N*_1_,…,*N_n_*)),

and take ctx = sid as the session context to which the Round 1 and Round 2 signatures, and all key derivation, are bound. This binding reduces the risk of unknown-key-share and cross-protocol attacks. The per-session base point is then given by Equation (6)

(6) G^*^ = 𝗁𝖺𝗌𝗁_𝗍𝗈_𝖼𝗎𝗋𝗏𝖾(𝗌𝗂𝖽),

using the RFC 9380 P-256 SSWU suite with the protocol’s fixed suite identifier and domain-separation tag [22]. The suite is deterministic and does not use trial-and-error rejection sampling. If the suite reports an internal expansion failure, or if the returned value is not a valid non-identity P-256 point (particularly if G^*^ = 𝒪), the session aborts. Because sid includes the fresh nonces, G^*^ is per-session. Its input is public, so G^*^ leaks nothing about any secret. P-256 has prime order and cofactor one, so any non-identity output is a generator of 𝔾; the implementation nonetheless relies on the suite guarantees and validates the returned point.

For reproducibility, the reference implementation pins the following concrete values. The protocol suite identifier is the ASCII string “PDKE-G-v1;P-256;XMD:SHA-256_SSWU_RO_;ECDSA-P256;HKDF-SHA256;AES-256-GCM”. The per-session base point is produced by the RFC 9380 random-oracle suite P256_XMD:SHA-256_SSWU_RO_ with the domain-separation tag “PDKE-G-v1_XMD:SHA-256_SSWU_RO_” (hexadecimal 50444b452d472d76315f584d443a5348412d3235365f535357555f524f5f), and the byte string supplied to hash_to_curve is “PDKE-G-v1;G*;” concatenated with the session identifier sid. Known-answer test vectors for the canonical descriptor, for credential derivation from a fixed test seed through the signing scalar and public key, and for a deterministic three-party transcript are provided in the reproducibility package.

### 4.4. Protocol Description

**Figure 3:**
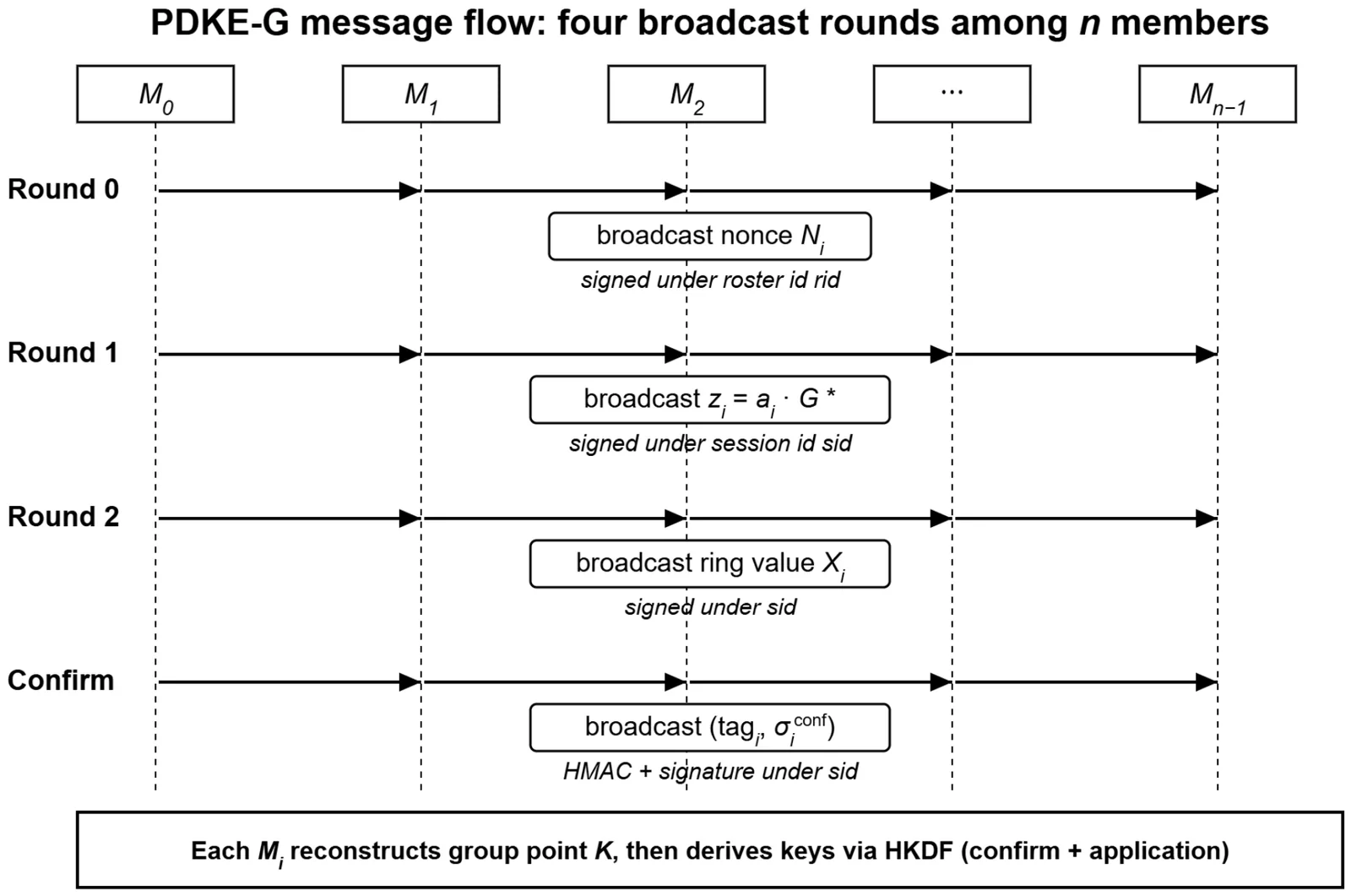
PDKE-G message flow: three broadcast rounds among the n members (nonce, contribution, ring value), each signed and bound to the roster or session identifier; every member then reconstructs the group point and derives the confirmation and application keys, after which a fourth signed-confirmation round binds the derived confirmation key.

The protocol has three broadcast rounds followed by a key-confirmation round (Figure 3). Members follow the canonical roster order and are indexed cyclically, so *U_n_*_+1_ = *U*_1_ and *U*_0_ = *U_n_*; indices below are taken modulo *n*. Every signature is computed over the canonical encoding of its message tuple: the notation Sig(*d_i_*,Encode(⋅)) below is explicit, and the parentheses inside Encode never abbreviate raw concatenation.

**Round 0 (Nonces):** Each *U_i_* samples a fresh 256-bit nonce $N_{i}\overset{R}{\leftarrow }\{0,1{\}}^{256}$ and broadcasts (*U_i_*,*N_i_*) with Sig(*d_i_*,Encode(“R0”,*i*,rosterID,*N_i_*)). A member MUST reject duplicate or equivocating nonce messages for the same index *i*, and MUST verify that the claimed participant identifier and public key occupy position *i* in the canonical roster. Every member verifies the others’ signatures, checks that the roster holds exactly *n* distinct registered public keys (aborting on any duplicate), and then forms sid and G^*^ as in Section 4.3.

**Round 1 (Contributions):** Each *U_i_* samples an ephemeral scalar $a_{i}\overset{R}{\leftarrow }Z_{q}^{*}$, computes *z_i_* = *a_i_* G^*^, and broadcasts *z_i_* with Sig(*d_i_*,Encode(“R1”,*i*,ctx,*z_i_*)).

**Round 2 (Ring Values):** Each *U_i_* computes and broadcasts

(7) *X_i_* = *a_i_* (*z_i_*_+1_ − *z_i_*_−1_),

signed as Sig(*d_i_*,Encode(“R2”,*i*,ctx,*X_i_*)), where *z_i_*_+1_ − *z_i_*_−1_ denotes elliptic-curve point subtraction.

**Acceptance Rules:** On receipt, a member MUST validate the canonical encoding and curve membership of every *z_i_* and *X_i_*, MUST reject *z_i_* = 𝒪 or *X_i_* = 𝒪 (the point at infinity), MUST reject any duplicate or equivocating message for the same (sid,round,*i*), and MUST verify the ECDSA signature under the claimed *Q_i_*. A member proceeds only if all *n* contributions in a round are accepted.

**Key Computation:** Each *U_i_* reconstructs the group point (Equation (8)):

(8) $$K\;=\;n\;a_{i}\;z_{i-1}\;+\;\sum_{t=0}^{n-2}\left(n-1-t\right)\;X_{\;i+t}.$$

The coefficients on the *X_i_*_+_*_t_* are chosen precisely so that the differences *e_i_* − *e_i_*_−1_ telescope into the ring sum $\sum_{j}a_{j}a_{j+1}$, as proved in Lemma 1. If *K* = 𝒪, the session key is undefined and the member MUST abort and restart; in an honest execution with independently sampled ephemeral scalars this occurs only with negligible probability, and the abort rule handles it without assuming any particular distribution on *K*. Otherwise, with Encode(*K*) the canonical encoding of the full point *K* (which distinguishes *K* from −*K*) and

(9) *τ* = 𝖲𝖧𝖠-256(𝖤𝗇𝖼𝗈𝖽𝖾(𝖼𝗍𝗑,*z*_1_,…,*z_n_*,*X*_1_,…,*X_n_*)),

key material is derived by fully specified HKDF-SHA-256, using distinct labels so that the confirmation and application keys are cryptographically domain-separated (and, under the PRF security of HKDF, computationally indistinguishable from independent pseudorandom keys),

(10) 𝖯𝖱𝖪 = 𝖧𝖪𝖣𝖥-𝖤𝗑𝗍𝗋𝖺𝖼𝗍(salt = 𝖼𝗍𝗑, IKM = 𝖤𝗇𝖼𝗈𝖽𝖾(*K*)),

(11) *K*_conf_ = 𝖧𝖪𝖣𝖥-𝖤𝗑𝗉𝖺𝗇𝖽(𝖯𝖱𝖪, 𝖤𝗇𝖼𝗈𝖽𝖾(“PDKE-G confirmation”,𝖼𝗍𝗑,*τ*), 32),

(12) *K*_app_ = 𝖧𝖪𝖣𝖥-𝖤𝗑𝗉𝖺𝗇𝖽(𝖯𝖱𝖪, 𝖤𝗇𝖼𝗈𝖽𝖾(“PDKE-G application key”,𝖼𝗍𝗑,*τ*), 32).

**Key Confirmation and Subsequent Data Protection:** A shared MAC alone cannot attribute a confirmation to a specific member, since every member holds *K*_conf_; we therefore combine key-possession evidence with a signature. Each *U_i_* computes tag*_i_* = HMAC-SHA-256(*K*_conf_,Encode(“confirm”,*i*,ctx,*τ*)) and broadcasts $\left({\mathrm{tag}}_{i},\;\sigma_{i}^{\mathrm{conf}}\right)$ with $\sigma_{i}^{\mathrm{conf}}=\mathrm{Sig}\left(d_{i},\mathrm{Encode}\left(\mathrm{“CONF”},i,\mathrm{ctx},\tau ,{\mathrm{tag}}_{i}\right)\right)$. A member accepts only after receiving exactly one valid pair for every roster index, verifying tag*_i_* in constant time and $\sigma_{i}^{\mathrm{conf}}$ under *Q_i_*, and confirming that all accepted messages bind the identical ctx and *τ*. The full 32-byte HMAC-SHA-256 tag is transmitted; truncation is not permitted. The MAC demonstrates possession of the session key material, while the signature provides sender attribution, so one insider cannot issue confirmations on behalf of other roster members. The suite identifier fixes the MAC algorithm alongside the curve, signature, KDF, and cypher.

**Broadcast Model:** A logical broadcast is implemented by sending the same message over point-to-point links in an adversarial network; message authentication is provided by the protocol signatures, and no ideal authenticated or consistent-broadcast primitive is assumed, following the network model of Katz–Yung [5]. A corrupt participant may therefore equivocate, sending different signed messages to different recipients. Such equivocation is detected before acceptance because each signed confirmation binds the complete transcript hash *τ*: honest members holding different transcripts require incompatible confirmation signatures, and no honest member accepts unless it receives, from every roster member, exactly one valid confirmation for its own identical (ctx,*τ*). The authentication analysis adopts this adversarial network model.

Subsequent group data is protected with AES-256-GCM [27] under *K*_app_. We require 3 ≤ *n* ≤ 2^32^ − 1 and 1 ≤ *i* ≤ *n*. Because a single key is shared by all senders, nonce uniqueness across senders MUST be enforced. Each sender uses a 96-bit nonce *N_i_*_,_*_c_* = I2OSP(*i*,4) ∥ I2OSP(counter*_i_*_,_*_c_*,8), where the canonical index *i* (fitting in 32 bits by the bound above) partitions the nonce space per sender and the 64-bit counter never repeats under *K*_app_, together with AAD = Encode(sid,*i*,counter*_i_*_,_*_c_*,message-type). Here the 4-octet I2OSP(*i*,4) inside the nonce is a domain-specific encoding used only for the AEAD nonce; in protocol transcripts Encode(*i*) is unchanged and uses the general 8-octet integer field.

The key MUST be renewed before any counter wraps. If a sender loses its persistent counter state (for example after a restart), the current group key MUST be retired and a fresh PDKE-G session MUST be executed before that sender transmits further data; this avoids any reliance on an under-specified epoch mechanism. Finally, AES-GCM under the shared group key authenticates a message as originating from some group member but does not provide sender attribution against malicious insiders; applications requiring sender attribution MUST additionally use per-sender signatures or sender-specific keys.

**Ephemeral-State Erasure:** After a session accepts or aborts, each party MUST securely erase its ephemeral scalar *a_i_* and all ephemeral Diffie–Hellman states, retaining only the derived application key *K*_app_ for as long as the application needs it. This erasure is required for the forward-secrecy claim: the security model of Section 5.1 provides no state-reveal query for the ephemeral state once erased, so a later corruption of the long-term signing key *d_i_* exposes nothing about a past *K*_app_.

### 4.5. Correctness

All scalar additions and multiplications in this proof are performed in ℤ*_q_*. We use 1-based indices 1,…,*n* taken modulo *n*, with the convention *U_n_*_+1_ = *U*_1_, *U*_0_ = *U_n_*, and set *e_j_* = *a_j_a_j_*_+1_ mod *q*.

**Lemma** **1** (Agreement)**.** *If all members follow the protocol, every member computes the same group point:*

(13) $$K\;=\;\left(\sum_{j=1}^{n}a_{j}a_{j+1}\right)\;G^{*}\;=\;\left(a_{1}a_{2}+a_{2}a_{3}+\ldots +a_{n}a_{1}\right)\;G^{*}.$$

**Proof.**  Since *z_i_* = a_i_G^*^, point subtraction gives *X_i_* = *a_i_*(*z_i_*_+1_ − *z_i_*_−1_) = (*a_i_a_i_*_+1_ − *a_i_*_−1_*a_i_*) G^*^ = (*e_i_* − *e_i_*_−1_) G^*^, and *n* *a_i_* *z_i_*_−1_ = (*n* *e_i_*_−1_) G^*^. Every summand in the key formula (Equation (8)) is thus a scalar multiple of the common generator G^*^, so the reconstructed point is *K* = *c_i_* G^*^ where, factoring out G^*^, the scalar coefficient is

(14) $$c_{i}\;=\;n\;e_{i-1}\;+\;\sum_{t=0}^{n-2}\gamma_{t}\;\left(e_{i+t}-e_{i+t-1}\right)\;\in \;Z_{q},\;\;\gamma_{t}=n-1-t.$$

Rearranging the telescoping sum with *γ_t_* − *γ_t_*_+1_ = 1, *γ_n_*_−2_ = 1, and *γ*_0_ = *n* − 1,

(15) $$\sum_{t=0}^{n-2}\gamma_{t}\;\left(e_{i+t}-e_{i+t-1}\right)\;=\;\sum_{t=0}^{n-2}e_{i+t}\;-\;\left(n-1\right)\;e_{i-1}.$$

The indices {*i*,*i* + 1,…,*i* + *n* − 2} mod *n* range over every residue except *i* − 1, so $\sum_{t=0}^{n-2}e_{i+t}=\left(\sum_{j=1}^{n}e_{j}\right)-e_{i-1}$. Substituting,

(16) $$c_{i}\;=\;n\;e_{i-1}+\sum_{j=1}^{n}e_{j}-e_{i-1}-\left(n-1\right)\;e_{i-1}\;=\;\sum_{j=1}^{n}e_{j},$$

which is independent of the index *i*. Therefore, every member reconstructs the same point $K=\left(\sum_{j}e_{j}\right)\;G^{*}$, and hence the same transcript *τ* and the same derived keys, *K*_conf_ and *K*_app_. □

The reference implementation confirms Lemma 1 empirically: across group sizes *n* = 3,…,50 every member derives an identical application key in every run.

### 4.6. Round, Communication, and Computation Complexity

The protocol uses a fixed number of rounds independent of *n*: three broadcast rounds for the authenticated key-exchange core plus one confirmation round, four in total. Counting one broadcast per member per round, the core exchange uses 3*n* messages and the complete protocol including confirmation uses 4*n* messages, both linear in the group size. For comparison, establishing keys by running the two-party PDKE protocol over all pairs uses mpairn2 messages; taking *m*_pair_ = 4 for a like-for-like full protocol with confirmation on both sides gives 2*n*(*n* − 1) = *Θ*(*n*^2^) messages, and it yields only pairwise keys rather than a single group key.

The empirical comparison includes confirmation on both sides. These are logical-broadcast counts, one per member per round, at which level PDKE-G is linear (4*n*) and the pairwise baseline is quadratic (2*n*(*n* − 1)). The picture changes when logical broadcasts are realized over point-to-point links, as our adversarial network model assumes. Delivering each of PDKE-G’s 4*n* logical broadcasts to the other *n* − 1 members entails 4*n*(*n* − 1) recipient transmissions, whereas the all-pairs baseline, whose messages are already point-to-point, requires only mpairn2=2nn−1 transmissions. Both are *Θ*(*n*^2^), but the constants differ: under this counting convention PDKE-G sends about twice as many raw transmissions, so its advantage is not a smaller transmission count.

The advantages are that PDKE-G completes in a constant number of logical rounds independent of *n*, and that it establishes a single contributory group key rather than n2 separate pairwise keys; on any network that does offer a native broadcast or multicast primitive, its 4*n*-versus-2*n*(*n* − 1) logical-message advantage is additionally realized at the transmission level.

The per-member computation is not independent of *n*. Each member generates four ECDSA signatures (for rounds R0, R1, R2 and the confirmation), verifies about 4(*n* − 1) peer signatures, computes one confirmation tag and verifies *n* − 1 peer confirmation tags, performs a constant number of full-size scalar multiplications for its own ephemeral value, and evaluates the Burmester–Desmedt combination, whose *n* − 1 scalar coefficients are small. Per-member work is therefore dominated by the 4(*n* − 1) signature verifications and is *O*(*n*); the total signature-verification work across the group is *O*(*n*^2^). The measured per-member time grows linearly with *n*, consistent with this analysis; the measurements, together with a summary table of rounds, messages, and per-member operations versus *n* and the details of the experimental environment, are reported in the evaluation.

## 5. Security Analysis

We analyze PDKE-G under a single, explicit adversary, but we separate two security goals into two experiments, because they hold under different corruption regimes and the earlier draft conflated them.

### 5.1. Model, Quantities, and Two Security Experiments

The model follows the indistinguishability tradition for authenticated key exchange of Bellare and Rogaway [28], with the session-oriented refinements of Canetti and Krawczyk [29], specialized to the group setting and to insider security along the lines of Katz and Shin [11].

**Parties, Sessions, and Quantities:** A fixed set of *P* registered parties each hold a long-term signing key *d_i_* with certified public key *Q_i_*, derived as in Section 4.2. A *session oracle* $\Pi_{i}^{t}$ is instance *t* of party *U_i_*; there are *q_s_* activated session oracles in total, and a single protocol run of a roster of size *n* activates *n* of them. We write *Q_N_* for the total number of nonces sampled by honest sessions (so *Q_N_* ≤ *q_s_*), 𝓁 = 256 for the nonce length (which equals the SHA-256 output length used for sid), *q_h_*_2_*_c_* for the number of queries to the hash_to_curve random oracle, and *q_H_* for the total number of SHA-256 evaluations, both honest and adversarial. These quantities are used with exactly these meanings throughout; *P* (all registered parties) is kept distinct from *n* (the roster size of one session), and the two hash oracles are counted separately by *q_h_*_2_*_c_* and *q_H_*.

**Adversary**: The adversary 𝒜 is probabilistic polynomial-time and controls the network exactly as in Section 4.4: a logical broadcast is realized over point-to-point links with no ideal authenticated or consistent-broadcast primitive, and 𝒜 may read, drop, reorder, replay, inject, and (for corrupt parties) equivocate. It interacts through $\mathrm{Send}\left(\Pi_{i}^{t},m\right)$, Execute({*U_i_*}), $\mathrm{Reveal}\left(\Pi_{i}^{t}\right)$ (returns a completed session’s application key), Corrupt(*U_i_*) (returns the long-term key *d_i_*), and a single $\mathrm{Test}\left(\Pi_{i}^{t}\right)$. Consistent with the erasure rule stated above, there is no state-reveal query for ephemeral state: once a session accepts or aborts, its *a_i_* is erased and unavailable to 𝒜.

**Partnering:** We distinguish two matching relations, because the confirmation round separates them. A session of *U_j_* is a *matching running session* of $\Pi_{i}^{t}$ if it reached its confirmation step holding the same sid = ctx (which by Section 4.3 binds the version, suite, group size, the full roster of public keys, and every nonce), the same transcript hash *τ*, and the same participant set; it is a *matching accepting session* if it additionally accepted. Two sessions are *partnered* when each is a matching running session of the other. Mutual acceptance is not guaranteed to a session over a fully adversarial network, since the adversary may withhold a needed confirmation from one honest party; the authentication goal below is therefore stated in terms of a unique matching running session, which is the achievable and standard form.

**Test Query and Winning Condition:** At the start the challenger samples $b\overset{R}{\leftarrow }\{0,1\}$. The single $\mathrm{Test}\left(\Pi_{i}^{t}\right)$ query is allowed only on a session that has already *accepted* and is AKE-fresh; it returns the real application key *K*_app_ if *b* = 1, and an independent uniformly random string of the same length (32 bytes) if *b* = 0. After Test, Reveal on the Test session or on any matching session is forbidden, and any action that would make the Test session non-fresh causes the experiment to output 0. At the end, 𝒜 outputs a guess *b*′, and ${\mathrm{Adv}}_{PDKE\text{-}G}^{\mathrm{ake}}\left(A\right)=2\;\left|\mathrm{Pr}\left[b^{\prime }=b\right]-1/2\right|$.

**Experiment 1 (AKE with Forward Secrecy):** The Test session and its matching sessions must be *AKE-fresh*: no Corrupt(*U_i_*) is issued against any member of the Test roster *before* that session accepts, and neither the Test session nor a matching session is Revealed. Corruption of long-term keys after acceptance is permitted; the resulting guarantee is post-accept long-term-key forward secrecy against an active network adversary under secure erasure, and it is well defined precisely because ephemeral state is erased.

**Experiment 2 (Injective Authentication Under Insider Corruption):** Here a coalition *C* ⊂ {*U*_1_,…,*U_P_*} may be Corrupted before or during execution, so 𝒜 holds every *d_j_* with *U_j_* ∈ *C*. The goal is *injective agreement restricted to honest peers*: for every honest accepting session $\Pi_{i}^{t}$ with *U_i_* ∉ *C*, and every peer *U_j_* ∉ *C* that $\Pi_{i}^{t}$ believes participated, there is a unique matching running session of *U_j_* (one that reached its confirmation step on the same (ctx,*τ*)). No agreement is claimed with a corrupt peer *U_j_* ∈ *C*: since 𝒜 legitimately holds *d_j_*, it may act as *U_j_*, and demanding a partner there would be unsatisfiable. Mutual acceptance is not claimed either, for the reason given under Partnering. This scoping is what makes the guarantee achievable, and it is the sound reading of “insider resistance”.

### 5.2. Cryptographic Assumptions

**Assumption** **1.** *Decisional Diffie–Hellman (DDH) in 𝔾, advantage ${\mathrm{Adv}}_{G}^{\mathrm{ddh}}$.*

**Assumption** **2.** *SUF-CMA of the instantiated signature Σ—ECDSA/P-256 under deterministic RFC 6979 signing, strict low-S verification, and canonical 64-byte encoding—with advantage ${\mathrm{Adv}}_{\Sigma }^{\mathrm{suf}}$. Strong unforgeability is the notion the Katz–Yung compiler requires. Deterministic signing (RFC 6979 [26]), canonical encoding, and strict low-S verification remove the encoding ambiguity and the direct (r, n − s) high-S variant—an honest signer emits a single signature per message—but they do not by themselves reduce SUF-CMA to EUF-CMA, because ECDSA verification still accepts other valid signatures for the same message and key that arise from different nonces.*

We therefore retain SUF-CMA as an explicit cryptographic assumption rather than deriving it from EUF-CMA. Consequently, the concrete security theorem is conditional on this explicit SUF-CMA assumption; RFC 6979 and low-*S* verification do not themselves establish it, and a deployment that requires a directly published guarantee may instantiate the compiler with a signature scheme carrying a proven SUF-CMA bound.

**Assumption** **3.** *KDF security of HKDF as an Extract-then-Expand construction: for key material drawn as the encoding of a uniform non-identity element of 𝔾_nz_ (the distribution of the randomized group secret used in the proof) and a public salt, HKDF-Extract yields a pseudorandom key PRK, and HKDF-Expand is a PRF; moreover, under distinct info labels the outputs K_conf_ and K_app_ are jointly indistinguishable from independent random keys (multi-output KDF security).*

We write the combined advantage ${\mathrm{Adv}}_{\mathrm{HKDF}}^{\mathrm{kdf}}$; this is the property established for HKDF as a computational extractor followed by a PRF [7].

**Assumption** **4.** *Random-oracle hash_to_curve: the RFC 9380 SSWU map is modelled as a random oracle onto generators of 𝔾, counted by q_h__2__c_*.

**Assumption** **5.** Credential pseudorandomness (HMAC as a PRF keyed by the secret seed): the extraction k_bio,__i_ equals HMAC-SHA-256(r_i_,D_i_) by the definition of HKDF, with the secret seed r_i_ in the HMAC key position; we assume the complete credential derivation of Section 4.2 is pseudorandom, and write ${\mathrm{Adv}}_{\mathrm{cred}}^{\mathrm{prf}}$ for the single-user advantage of distinguishing the derived signing scalar d_i_ from a uniform scalar in $Z_{q}^{*}$.

This one advantage bounds the whole path *r_i_* → *k*_bio,_*_i_* → HKDF-Expand → *d_i_*: it follows from the PRF security of HMAC-SHA-256 under the uniform secret seed, the PRF security of HKDF-Expand at the fixed scalar-derivation label, and the negligible probability of counter exhaustion, while the rejection sampling of Section 4.2 maps a uniform expansion output to a scalar that is exactly uniform on [1, *q* − 1] conditioned on acceptance. Hence, *k*_bio,_*_i_* is pseudorandom and the signing scalar *d_i_* is computationally indistinguishable from a uniform signing key, whence Assumption 2 applies to *Q_i_*.

This advantage is charged explicitly in Theorems 1 and 2 (with a hybrid over the *P* registered parties) rather than being silently absorbed. We rely on this PRF assumption rather than on the min-entropy of *r_i_* alone: standard HKDF analysis treats the IKM as the entropy-bearing input and the salt as a non-secret randomizer, so the security here comes specifically from keying HMAC with the secret seed.

**Assumption** **6.** SHA-256 session binding: the SHA-256 function used to form rosterID, sid, and the transcript hash τ is modelled as a random oracle, counted separately by q_H_; consequently, it is collision-resistant, so distinct honest nonce vectors yield distinct sid except with birthday probability, and sid^*^ formed from at least one unrevealed uniform 256-bit nonce is computationally unpredictable before that nonce is released.

This assumption supports both the session-identifier uniqueness used in *G*_0_ → *G*_1_ and the early-query bound of the random-oracle programming in *G*_2_ → *G*_3_; a reader who prefers a standard-model statement may instead read it as SHA-256 collision resistance together with an explicit unpredictability advantage, added to the bounds with the same normalization.

The empirical study motivates Assumption 5 but does not evidence it: the study shows why a secret seed is needed, whereas the PRF security of HMAC and HKDF on which Assumption 5 rests is a standard cryptographic assumption that the structural study neither establishes nor tests. The selection space of known proteins is only about 2^27.7^, so if *r_i_* were public the credential, and hence *d_i_*, would be recoverable by an offline search over candidate proteins under the known key, and Assumption 2 would fail.

The secret 128-bit seed both keys the PRF and makes exhaustive seed recovery require approximately 2^127^ trials on average (up to 2^128^ in the worst case), with a *q*-guess adversary succeeding with probability at most about *q*/2^128^ apart from the HMAC-PRF advantage. Protocol identity is anchored by the certified public key and this seed, not by any claim of universal collision-freedom of protein geometry: the geometry is a distinct member-specific input, and the 519-structure study reports no collisions in that sample rather than a universal guarantee.

PDKE-G is therefore not a password-authenticated exchange. Because *Q_i_* is public and the derivation from (*r_i_*,*D_i_*) to *d_i_* is deterministic, an adversary can validate guessed seed–descriptor pairs entirely offline by recomputing *d_i_G* and comparing it with *Q_i_*; there is no one-candidate-per-session restriction. For a fixed target and a known descriptor, *q* seed guesses succeed with probability at most about *q*/2^128^, apart from the credential-PRF advantage, so only the seed entropy makes offline recovery infeasible.

### 5.3. Symbolic Verification in ProVerif

We modelled the three-party instantiation in the applied pi-calculus and verified it with ProVerif [8]. Three queries returned true: secrecy of the session key, which survives revealing the long-term keys in a later phase and so witnesses forward secrecy; injective mutual authentication, the query inj-event(acceptAB) ⇒ inj-event(runB); and injective authenticity of another member’s contribution, the query inj-event(acceptAC) ⇒ inj-event(runC). The companion four-party model verifies the same three queries, confirming that the properties are not an artefact of the smallest group size.

Three points fix the scope of this evidence. First, the abstraction map: the symbolic context is ctx = (*N_A_*,*N_B_*,*N_C_*), an abstraction of the concrete sid of Section 4.3, which additionally binds the suite identifier, the group size, and the roster of public keys. The symbolic model represents the roster implicitly through the distinct long-term keys of the named participants and the injective pairing of signatures to ctx, and the concrete binding of suite and roster is argued concretely rather than re-encoded symbolically.

Second, ProVerif captures multiplicative Diffie–Hellman and not the additive exponent arithmetic of the Burmester–Desmedt ring, so the group key is represented through its Diffie–Hellman core. The secrecy of the *n*-party ring key for general *n* is established computationally in Section 5.4, and this symbolic result is corroborating evidence for the three-party (and four-party) message logic, not a substitute for that proof, nor a claim about general *n* or about every form of member tampering. Third, an earlier version of the model without the Round 0 nonce exchange satisfied only non-injective agreement, that is it was replayable; adding the nonce round that binds ctx into every signature is what upgraded the guarantee to injective agreement, which shows Round 0 to be necessary for injective agreement in the studied ProVerif abstraction and counter-model.

ProVerif confirms this contrast directly: on the without-Round-0 counter-model, the non-injective query holds but the injective query is false, returned with an explicit replay trace in which one signed contribution is accepted in two distinct sessions, whereas on the Round-0 models all injective queries hold. All six queries were discharged by ProVerif 2.05, establishing for each that every accept event is injectively associated with a distinct preceding run event. The tool version (2.05), the model files for *n* = 3 and *n* = 4, the without-Round-0 counter-model that exhibits the replay, the exact queries, and the tool output are included in the reproducibility package so the result can be reproduced; an unbounded- *n* symbolic treatment in Tamarin [17] is left as future work.

### 5.4. Key Indistinguishability with Forward Secrecy

PDKE-G is the passively secure Burmester–Desmedt (BD) protocol over the per-session generator *g* = G^*^, wrapped by the Katz–Yung (KY) authentication compiler whose signatures are the protein-derived identities of Section 4.2, with session keys derived by HKDF. We prove Theorem 1 by making this correspondence explicit and composing two published reductions with a few explicit game hops. Table 1 summarises the correspondence between PDKE-G elements and their published counterparts.

The published Katz–Yung compiler theorem [5] applies to concurrent sessions, an arbitrary number of parties, and forward secrecy, assuming only that the underlying protocol is secure against a passive adversary; the passive security of BD under DDH is likewise a published result [4,5]. We invoke Katz–Yung for the qualitative compiler theorem, that authentication reduces active security to passive security, and we give a protocol-specific compositional bound for PDKE-G, whose Burmester–Desmedt term is the published passive advantage invoked as a black box (the corollary below), not a re-derived DDH constant of our own.

The bound of Theorem 1 is therefore not quoted verbatim from the Katz–Yung theorem, whose original statement carries its own session and compiler-collision terms; instead, each of its terms follows from the explicit games presented below, with the passive BD-under-DDH result invoked inside the DDH hop. Two instantiation points are made explicit. First, PDKE-G runs over the per-session generator *g* = G^*^ = hash_to_curve(sid); once hash_to_curve is modelled as a random oracle and the oracle programming of *G*_2_ → *G*_3_ is carried out, *g* is a uniformly random generator of 𝔾 compatible with the DDH reduction, so the published BD-under-DDH result applies over *g*.

Second, the compiler’s signatures are keyed by the protein-derived identities, which, by Assumption 5, are indistinguishable from uniform signing keys. Our contribution at this step is the correspondence and the concrete instantiation, not a new reduction for BD or for the compiler.

Throughout we use one normalization: every hop is bounded on the difference |Pr[*S_i_*] − Pr[*S_i_*_+1_]|, where *S_i_* is the event *b*′ = *b* in Game *G_i_*, and the final advantage is recovered once, at the end, through Adv^ake^ = 2 |Pr[*S*_0_] − 1/2|.

**Theorem** **1** (AKE with Forward Secrecy)**.** *In the AKE-fresh experiment of Section 5.1, for every probabilistic polynomial-time* 𝒜 *there exist probabilistic polynomial-time* ℬ_0_,ℬ_1_,ℬ_2_,ℬ_3_ *such that, writing ε_coll_* = (*Q_N_*(*Q_N_* − 1) + *q_H_*(*q_H_* − 1))/2^𝓁^ *for the combined nonce and SHA-256 birthday terms,*

(17) $${\mathrm{Adv}}_{\mathrm{PDKE}-G}^{\mathrm{ake}}\left(A\right)\;\leq \;2P\;{\mathrm{Adv}}_{\mathrm{cred}}^{\mathrm{prf}}\left(B_{0}\right)+2P\;{\mathrm{Adv}}_{\Sigma }^{\mathrm{suf}}\left(B_{1}\right)+2q_{s}\;{\mathrm{Adv}}_{\mathrm{BD}}^{\mathrm{ke}-\mathrm{fs}}\left(B_{2};t,1\right)+2\;{\mathrm{Adv}}_{\mathrm{HKDF}}^{\mathrm{kdf}}\left(B_{3}\right)+\varepsilon_{\mathrm{coll}}+\frac{q_{H}+q_{h2c}}{2^{l-1}}+\frac{2}{q},+\varepsilon_{\mathrm{abort}}$$

*where* ${\mathrm{Adv}}_{\mathrm{cred}}^{\mathrm{prf}}$ *is the single-user advantage against HMAC-SHA-256 as a PRF keyed by the secret seed (Assumption 5); if a multi-user credential-PRF advantage is used instead, it absorbs the hybrid and the explicit factor P on that term is dropped. Here* ${\mathrm{Adv}}_{\mathrm{BD}}^{\mathrm{ke}-\mathrm{fs}}\left(B_{2};t,1\right)$ *denotes the published passive single-Execute key-establishment advantage of the Burmester–Desmedt core under DDH, for the single-Execute adversary produced in the DDH hop, invoked as a black box; the Corollary 1 below substitutes it by the raw DDH advantage with the constant of the published analysis.*

**Proof.**  Let *S_i_* be the event *b*′ = *b* in *G_i_*; by the triangle inequality $\left|\mathrm{Pr}\left[S_{0}\right]-1/2\right|\leq \sum_{i=0}^{3}\left|\mathrm{Pr}\left[S_{i}\right]-\mathrm{Pr}\left[S_{i+1}\right]\right|+\left|\mathrm{Pr}\left[S_{4}\right]-1/2\right|$, and we bound each term and multiply by 2 at the end.

*G*_0_ → *G*_1_ **(nonce and session-identifier uniqueness).** Abort on either of two events: two honest sessions sample the same nonce, or two distinct SHA-256 inputs among the rosterID, sid, and *τ* evaluations collide. By the standard birthday (union) upper bound over *Q_N_* nonces and, under Assumption 6, over the *q_H_* SHA-256 evaluations, |Pr[*S*_1_] − Pr[*S*_0_]| ≤ *Q_N_*(*Q_N_* − 1)/2^𝓁+1^ + *q_H_*(*q_H_* − 1)/2^𝓁+1^. Thereafter, except for matching session oracles of a single execution, which, by design, share the same nonces, distinct honest executions have distinct nonce vectors and, by SHA-256 collision resistance, distinct ctx and distinct sid.

*G*_1_ → *G*_2_ **(credential pseudorandomness and signature unforgeability, KY authentication step):** This hop has two stages. First, by Assumption 5, we replace each of the *P* registered parties’ protein-derived signing keys *d_i_* (and the corresponding public keys *Q_i_* = *d_i_G*) by uniformly random ECDSA signing keys; a hybrid over the *P* parties bounds the resulting distinguishing advantage by $P\;{\mathrm{Adv}}_{\mathrm{cred}}^{\mathrm{prf}}\left(B_{0}\right)$, where ${\mathrm{Adv}}_{\mathrm{cred}}^{\mathrm{prf}}$ is the single-user PRF advantage of HMAC-SHA-256 keyed by the secret seed (a multi-user credential-PRF advantage would absorb this hybrid and drop the factor *P*). Second, with the signing keys now uniform, we abort if any honest session accepts a message that verifies under an honest *Q_i_* but was not produced by *U_i_*; a further hybrid over the *P* registered parties turns this event into a SUF-CMA forgery.

Hence, $\left|\mathrm{Pr}\left[S_{2}\right]-\mathrm{Pr}\left[S_{1}\right]\right|\leq P\;{\mathrm{Adv}}_{\mathrm{cred}}^{\mathrm{prf}}\left(B_{0}\right)+P\;{\mathrm{Adv}}_{\Sigma }^{\mathrm{suf}}\left(B_{1}\right)$. This is the protocol-specific analogue of the Katz–Yung authentication step: after *G*_2_ every delivered flow accepted by an honest AKE-fresh session equals an honestly generated flow bound to that session’s ctx, so the active adversary is reduced to a passive one on the BD core. (No long-term key of the Test roster has been corrupted before acceptance, by AKE-freshness, so this hybrid is sound for the Test session.)

*G*_2_ → *G*_3_ **(embed DDH into the Test session, with random-oracle programming).** The reduction guesses the index t^*^ ∈ {1,…,*q_s_*} of the Test session, incurring the loss factor 1/*q_s_*, and sets the base point *g* = hash_to_curve(sid^*^) of that session to the generator of a DDH instance. The random oracle is programmed lazily, not in advance of the nonces. The Test session’s base point is the single value hash_to_curve(sid^*^), and sid^*^ is fixed only after every Round 0 nonce of that session has been broadcast and its accompanying signature verified. At that moment, and before this base point is first used in Round 1, the reduction sets hash_to_curve(sid^*^) = *g*, the generator of its DDH instance.

This assignment is consistent with the view of 𝒜 unless 𝒜 had already queried hash_to_curve at the point sid^*^ before that Round 0 message was released. Because sid^*^ commits to at least one honest, freshly sampled, high-entropy nonce that stays unrevealed until its signed Round 0 message is broadcast, no query made before that release can equal sid^*^ except by predicting that nonce, and distinct sessions use distinct nonces except with negligible collision probability; the programming therefore affects the Test session alone. Under Assumption 6, sid^*^ is the SHA-256 image of an input containing that unrevealed uniform 256-bit nonce, so 𝒜 can neither compute sid^*^ from the SHA-256 oracle (which would require querying the right preimage, with probability at most *q_H_*/2^𝓁^) nor guess it blindly at hash_to_curve (probability at most *q_h_*_2_*_c_*/2^𝓁^); the bad event is therefore at most (*q_H_* + *q_h_*_2_*_c_*)/2^𝓁^.

Modelling SHA-256 as a random oracle is exactly what makes sid^*^ unpredictable here, and it is the only place the assumption is used in this hop; distinct base points across sessions are not otherwise a security requirement, because sid, the signatures, and HKDF already bind each session, so no separate random-oracle-collision hop and no *q*^2^/*q* term arise. Conditioned on a correct guess and on the complement of the bad event, replacing the BD secret *K* of the Test session by a uniform $\tilde{K}\in G_{\mathrm{nz}}$ is bounded by the *published* passive key-establishment advantage of Burmester–Desmedt under DDH, which we denote ${\mathrm{Adv}}_{\mathrm{BD}}^{\mathrm{ke}-\mathrm{fs}}$ and invoke as a black box [4,5].

That published reduction embeds a DDH challenge into the ring and simulates the Burmester–Desmedt transcript {*z_i_*,*X_i_*} and group key, returning a $\tilde{K}$ uniform on 𝔾. The signatures and the active Send, Reveal, Corrupt, and Test queries are handled by the outer game hybrid already established in *G*_1_ → *G*_2_, not by the passive BD reduction. We do not re-derive its constant here; the Corollary 1 below records the published relation between ${\mathrm{Adv}}_{\mathrm{BD}}^{\mathrm{ke}-\mathrm{fs}}$ and the raw DDH advantage. One added statistical step is ours: moving to the accepted-session distribution, which conditions on every *X_i_* ≠ 𝒪 and on *K* ≠ 𝒪 by the abort rules of Section 4.4, costs total statistical distance at most *ε*_abort_ ≤ *n*/(*q* − 1) + 1/*q* since each of the n contribution checks aborts with probability 1/(*q* − 1) and $\mathrm{Pr}\left[\tilde{K}=O\right]=1/q$, so the total conditioning cost is exactly *ε*_abort_; because both rules are public functions of the transcript, the reduction enforces them identically in the real and simulated experiments.

Hence, $\left|\mathrm{Pr}\left[S_{3}\right]-\mathrm{Pr}\left[S_{2}\right]\right|\leq q_{s}\;{\mathrm{Adv}}_{\mathrm{BD}}^{\mathrm{ke}-\mathrm{fs}}\left(B_{2}\right)+\left(q_{H}+q_{h2c}\right)/2^{l}+1/q$, with the factor *q_s_* coming only from the Test-session guess. The embedding uses only ephemeral scalars, so it is unaffected by Corrupt queries issued *after* the Test session accepts; this is the source of forward secrecy, well defined only because the ephemeral state is erased (Section 4.4).

*G*_3_ → *G*_4_ **(key derivation).** With Encode(*K*) now the encoding of a uniform independent non-identity point, Assumption 3 (whose input distribution is exactly the encoding of a uniform element of 𝔾_nz_) replaces the HKDF outputs of the Test session by independent random keys; because *K*_conf_ and *K*_app_ use distinct info labels, the confirmation tags computed under *K*_conf_ and revealed on the network do not help distinguish *K*_app_, and $\left|\mathrm{Pr}\left[S_{4}\right]-\mathrm{Pr}\left[S_{3}\right]\right|\leq {\mathrm{Adv}}_{\mathrm{HKDF}}^{\mathrm{kdf}}\left(B_{3}\right)$.

In *G*_4_, the Test key is uniform and independent of *b*, so Pr[*S*_4_] = 1/2. Substituting the four bounds and multiplying by 2 gives the theorem, with 2 ⋅ *Q_N_*(*Q_N_* − 1)/2^𝓁+1^ = *Q_N_*(*Q_N_* − 1)/2^𝓁^, 2 ⋅ *q_H_*(*q_H_* − 1)/2^𝓁+1^ = *q_H_*(*q_H_* − 1)/2^𝓁^, 2 ⋅ (*q_H_* + *q_h_*_2_*_c_*)/2^𝓁^ = (*q_H_* + *q_h_*_2_*_c_*)/2^𝓁−1^, and 2 ⋅ 1/*q* = 2/*q*. □

**Corollary 1**  (Single-Execute DDH Instantiation)**.** *By the published passive-security analysis of the Burmester–Desmedt core under DDH [4,5], the black-box term satisfies* ${\mathrm{Adv}}_{\mathrm{BD}}^{\mathrm{ke}-\mathrm{fs}}\left(B_{2};t,1\right)\leq 4\;{\mathrm{Adv}}_{G}^{\mathrm{ddh}}\left(B_{2}\prime ;t\prime \prime \right)$ for a related distinguisher ℬ_2_′ in the single-Execute formulation (t,1) of Katz–Yung (Theorem 2 of [5], journal p. 104), whose single-Execute statement additionally retains a 2/|G| term; propagating it gives 8q_s*Adv_ddh + 4q_s/|G| rather than 8q_s*Adv_ddh alone, and the concrete bound further includes an explicit abort term eps_abort <= n/(q − 1) + 1/q from the X_i = O and K = O sampling and rejection rules. We take the single-Execute relation ${\mathrm{Adv}}_{\mathrm{BD}}^{\mathrm{ke}-\mathrm{fs}}\leq 4\;{\mathrm{Adv}}^{\mathrm{ddh}}$ *as the basis of the substitution. The distinguisher here is the passive single-Execute adversary produced in the DDH game hop, after the outer reduction has guessed the Test-session index, so the single-Execute part of the published Katz–Yung analysis applies with a single Execute query. Substituting the* $4\;{\mathrm{Adv}}_{G}^{\mathrm{ddh}}$ *relation into Theorem 1 replaces* $2q_{s}\;{\mathrm{Adv}}_{\mathrm{BD}}^{\mathrm{ke}-\mathrm{fs}}$ *by* $8q_{s}\;{\mathrm{Adv}}_{G}^{\mathrm{ddh}}+\frac{4q_{s}}{\left|G\right|}$, yielding a bound expressed purely in the DDH, signature, credential-PRF, and KDF advantages. The multi-session loss is already carried by the outer Test-session-guess factor in Theorem 1, so the inner Burmester–Desmedt reduction itself uses a single Execute query. The X_i = O and K = O rejection rules are public and decidable from the transcript alone, so the reduction applies them identically in the real and simulated experiments; they contribute no distinguishing advantage beyond the abort probability eps_abort <= n/(q − 1) + 1/q, which is the term now carried explicitly as the +eps_abort summand in the Theorem 1 bound (Equation (17)) and propagated into the corollary bound 8q_s*Adv_ddh + 4q_s/|G|. We state Theorem 1 in the compositional form so that any future sharpening of the published Burmester–Desmedt bound propagates directly.

### 5.5. Injective Authentication and Insider Resistance

**Theorem** **2** (Injective Agreement for Honest Peers)**.** *In the insider experiment of Section 5.1, for every honest accepting session* $\Pi_{i}^{t}$ *(U_i_ ∉ C) and every peer U_j_ ∉ C that* $\Pi_{i}^{t}$ *believes participated, there is, except with probability at most* $P\;{\mathrm{Adv}}_{\mathrm{cred}}^{\mathrm{prf}}+P\;{\mathrm{Adv}}_{\Sigma }^{\mathrm{suf}}+Q_{N}\left(Q_{N}-1\right)/2^{l+1}+q_{H}\left(q_{H}-1\right)/2^{l+1}$*, a unique matching running session of U_j_ on the same (ctx,τ), that is, a session of U_j_ that reached its confirmation step with this (ctx,τ).*

**Proof.**  We argue by rounds, because the flows are bound to different identifiers. The Round 0 signature of *U_j_* is over Encode(*“*R0”,*j*,rosterID,*N_j_*), binding the roster and *U_j_*’s nonce before ctx exists; the Round 1 and Round 2 signatures are over Encode(*“*R1”/*“*R2”,*j*,ctx,⋅); and the confirmation signature is $\sigma_{j}^{\mathrm{conf}}=\mathrm{Sig}\left(d_{j},\mathrm{Encode}\left(“\mathrm{CONF”},j,\mathrm{ctx},\tau ,{\mathrm{tag}}_{j}\right)\right)$. A contribution signature by itself establishes only message *origin*, so the agreement is derived from the confirmation. Since $\Pi_{i}^{t}$ accepts, it verified $\sigma_{j}^{\mathrm{conf}}$ under *Q_j_*; as *U_j_* ∉ *C*, its key *d_j_* is secret. By Assumption 5, we first replace the *P* protein-derived signing keys by uniform ECDSA keys, at cost $P\;{\mathrm{Adv}}_{\mathrm{cred}}^{\mathrm{prf}}$, so that the standard SUF-CMA assumption applies to the resulting keys; then, by SUF-CMA (hybrid over the *P* parties), *U_j_* itself produced this signature on this exact (ctx,*τ*,tag*_j_*), and hence ran a session that reached its confirmation step with this (ctx,*τ*).

Nonce uniqueness and SHA-256 collision resistance (the two birthday terms, under Assumption 6) make ctx unique to a single grouping, and an honest session emits exactly one confirmation, so that matching running session of *U_j_* is unique. The guarantee is a unique matching running session, not mutual acceptance: over a fully adversarial network 𝒜 may deliver *U_j_*’s confirmation to $\Pi_{i}^{t}$ while withholding some other member’s confirmation from *U_j_*, so *U_j_* need not itself accept; no number of additional rounds can force mutual acceptance against a network adversary that may drop messages, so it is not claimed.

No claim is made for *U_j_* ∈ *C*, whose key 𝒜 holds. □

**Corollary** **2** (Member-Tampering Resistance)**.** *Under Assumptions 2, 5, and 6, and except with the credential-PRF, signature-SUF, nonce-collision, and SHA-256-collision terms in the bound of Theorem 2, a corrupt coalition C cannot cause an honest session to accept a run whose roster lists an honest party U_j_ ∉ C as a participant unless U_j_ actually took part, producing its own signatures and confirmation for that ctx.*

**Proof.**  Acceptance requires, for the index of *U_j_*, a valid Round 0 to Round 2 signature chain and a valid confirmation under *Q_j_* bound to rosterID and ctx; producing any of these without *d_j_* is an SUF-CMA forgery. The member-tampering attack of Sun and Tian [6] uses corrupted members shared between two groups to make honest members of one group believe that uninvolved members of the other participated; the enabling capability, in either its two-group form or a single-group substitution variant, is to have a party accepted as a participant without its own genuine authenticated contribution. Against PDKE-G, the combination of roster and context binding, a per-member signature on every flow, and a confirmation over the shared transcript *τ*, prevents any honest non-participant from being inserted, which is exactly the capability this corollary rules out. □

We claim no more than this: Corollary 1 prevents impersonation or insertion of an honest non-participant; it does not by itself establish contributiveness or key-control resistance against every insider coalition, which are separate properties outside the present scope. An executable negative control illustrates this capability empirically.

The composition above makes each role clear. The Katz–Yung compiler provides the qualitative justification for one step, *G*_1_ → *G*_2_: for parties uncorrupted at the time of acceptance, authentication turns an active network adversary into a passive one, and our protocol-specific hybrid realizes this at the cost of $P\;{\mathrm{Adv}}_{\mathrm{cred}}^{\mathrm{prf}}+P\;{\mathrm{Adv}}_{\Sigma }^{\mathrm{suf}}$ under the single-user normalization, with the credential-PRF term arising because the same hybrid first replaces the protein-derived signing keys by uniform keys (Assumption 5). The quantitative bound of Theorem 1 is compositional, as established by the preceding games, with the passive Burmester–Desmedt advantage invoked as a published black box.

The compiler does not by itself yield the insider and injectivity guarantees of Theorem 2; those come from two design choices analyzed here, namely binding every signature to the roster and to ctx through the Round 0 nonce exchange, and giving each member a distinct protein-derived signing key rather than a shared credential, and are proved directly in Theorem 2.

**Corollary** **3** (Key Agreement Among Accepting Honest Peers)**.** *In any execution, including one containing corrupted roster members, if two honest sessions accept with the same roster and are matching on the session identifier and transcript hash, as required by Theorem 2, then they reconstruct the same group point K and derive identical confirmation and application keys K_conf and K_app.*

**Proof.**  Let two honest sessions hold the same session identifier and accept. Acceptance at a session requires, from every roster member, a confirmation tag that verifies under that session’s own confirmation key K_conf and is bound to the session identifier and the transcript hash. Suppose the two sessions had reconstructed different group points, and hence different K and different K_conf. Then each session would have accepted, under its own K_conf, the confirmation tags that the honest members computed under the other key; producing such cross-verifying tags is an existential forgery against HMAC-SHA-256, which, by its SUF-CMA security, occurs with probability at most q_s times the SUF-CMA advantage of HMAC-SHA-256. Except on that event, the two sessions agree on K_conf, on the transcript hash it authenticates, and therefore on the reconstructed point K; since the application key K_app is a deterministic HKDF function of K and the session identifier, the two sessions derive the identical K_app. This argument relies only on the unforgeability of the confirmation tags and does not invoke Lemma 1. It is agreement among accepting honest peers: it does not imply mutual acceptance, since the adversary may withhold a confirmation from another honest running session, nor does it claim agreement with a corrupt peer. □

### 5.6. Cryptanalysis: Attacks Considered and Scope

This section consolidates the concrete attacks considered against PDKE-G and their outcomes. Replay and cross-session reinterpretation are prevented by binding every contribution to the per-session context: the symbolic analysis confirms injective agreement for the three- and four-party instantiations, and the no-Round-0 counter-model exhibits an explicit replay trace in which a single signed contribution is accepted in two sessions, so the nonce round is necessary. Honest-member insertion in the member-tampering setting is defeated within the scope of Corollary 2 by the per-member certified signing keys, together with the roster and session binding; the executable negative control accepts none of 200 tampered runs, whereas the shared-credential baseline accepts all of them. Network impersonation of uncorrupted members is excluded by the per-member signatures under the Katz–Yung compiler.

Recovery of the long-term credential was analyzed directly. With the secret seed in the HMAC key position, recovering a signing key costs 2^128 work regardless of whether the input is the real geometry descriptor, a public accession string, or a constant; an ablation confirms this and shows that a variant without the secret seed is recovered by enumerating the public structure space, whose size is at most about 2^27.7. This is why the secret seed is an explicit part of the design rather than optional.

Binding integrity distinguishes the structure-derived descriptor from an ordinary public name. For brute-force secrecy the descriptor is interchangeable with an accession string or a constant, as above; what it adds instead is a content commitment to the member’s structure of record. Under the certificate attestation of Section 4.2, the member’s key is bound to SHA-256(D_i) with D_i = canon(structure), so a relying party that recomputes canon of the referenced public structure detects any substitution or repointing of that structure and the certificate fails. An accession string is only a pointer: the content behind it can be replaced without detection. A naive hash of the raw coordinate file is substitution-sensitive but not reproducible, since it changes under benign differences in coordinate precision or file encoding; the 0.01 Å canonicalization is what keeps the descriptor stable under such differences while remaining sensitive to genuine substitution. An executable control (Script S1; output in Text S1) accompanies the reproducibility package: for one key certified under a name binding, a raw-file-hash binding, and the canonical-descriptor binding, only the descriptor binding both accepts a sub-quantization re-expression of the enrolled structure and rejects a 0.1 Å single-residue substitution presented under the same name, whereas the name binding accepts the substitution and the raw-file binding rejects the benign re-expression. The binding is to a specific, publicly reproducible structure of record; it does not certify that the protein is biologically authentic.

The following guarantees are explicitly out of scope. The shared-key authenticated encryption used after key agreement attributes a message to some group member but not to a specific sender against malicious insiders; applications requiring sender attribution must add per-sender signatures or sender-specific keys. Mutual acceptance is not guaranteed over a network that can drop messages, and the construction is not a password-authenticated key exchange, so the secret seed must be high-entropy.

## 6. Implementation and Evaluation

This section reports a reference implementation of PDKE-G and four bodies of evidence produced with it: correctness across group sizes, an empirical study of the protein-derived credential over 519 real structures, executable and symbolic security evidence, and a performance and scalability measurement. Every experiment was executed on a standard Google Colab runtime, and the reference implementation, the ProVerif models, the entropy dataset, and the evaluation notebooks are collected in a reproducibility package, to be made available with the published article, so that all figures and tables can be regenerated end-to-end.

### 6.1. Reference Implementation and Correctness

**Figure 4:**
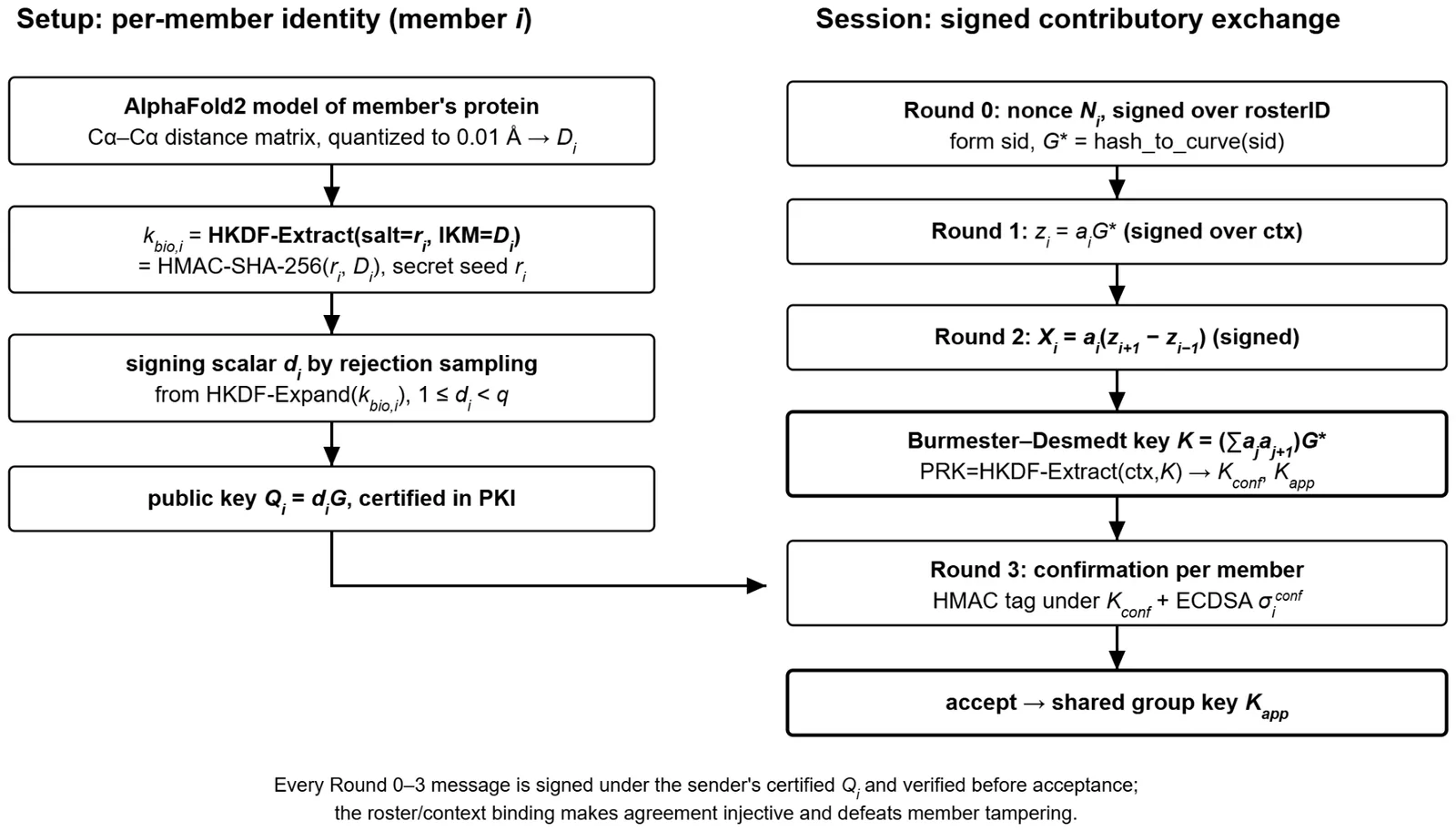
The PDKE-G protocol: per-member protein-derived identities feed a signed, session-bound Burmester–Desmedt exchange that yields a single contributory group key with a signed confirmation round.

The reference implementation realizes the full specification of Section 4 over the NIST P-256 curve: the RFC 9380 SSWU hash_to_curve map with the protocol’s domain-separation tag, ECDSA with deterministic (RFC 6979) nonces and low-*S* encoding, HKDF-SHA-256 for extraction and expansion, HMAC-SHA-256 for key confirmation, and AES-256-GCM for subsequent data protection. It implements the canonical length-prefixed encoding, the roster and session identifiers, the per-session base point, strict public-key and point validation, the abort rules including the rejection of *z_i_* = 𝒪, *X_i_* = 𝒪, and *K* = 𝒪, and the signed confirmation round. The pure-Python implementation is intended for reproducibility and functional evaluation, not production deployment; production systems should use audited cryptographic libraries. All measurements were taken on Google Colab; the flow of the protocol as implemented is summarized in Figure 4.

Correctness was verified by a self-test that runs the complete protocol at every group size of Table 2, namely, *n* ∈ {3,4,5,8,10,15,20,30,40,50}, with five independent repetitions per group size, using fresh per-member seeds and ephemeral randomness in every repetition. Each run checks that all *n* members reconstruct an identical application key, that the signed confirmation verifies for every member, and that the ancillary primitives behave as specified. Across all runs the SSWU map returned on-curve, deterministic outputs; ECDSA accepted valid signatures and rejected tampered ones; and every group exchange reported identical keys and successful confirmation, matching Lemma 1 (Equation (13)). The self-test reports ALL SIZES PASS, confirming the agreement property empirically over the full range of evaluated sizes.

### 6.2. Empirical Study of the Protein-Derived Credential

*Dataset:* To ground the descriptor of Section 4.2 in real data, we evaluated 519 predicted models from the AlphaFold Protein Structure Database [15] (the resolved download URL, model version, and file SHA-256 digest for every structure are recorded in the reproducibility package), drawn from seven reference proteomes: Saccharomyces cerevisiae (108), Escherichia coli K-12 (100), Homo sapiens (87), Caenorhabditis elegans (67), Drosophila melanogaster (65), Arabidopsis thaliana (52), and Mus musculus (40). For each proteome, we selected entries in ascending UniProt-accession order, retaining single-chain models whose resolved Cα count lies in [16, 500] (mean 307) and skipping entries with missing or malformed coordinate records.

The complete list of UniProt accessions and the per-structure statistics are provided in the reproducibility package as PDKE-G_entropy_normative_record.csv (the dataset of record; identical in content to the file earlier named PDKE-G_entropy_sample.csv), alongside the unmodified original tabulation PDKE-G_entropy_original_v4.csv and the verification tool used to regenerate them. For each model we built the canonical quantized upper-triangular Cα–Cα distance descriptor *D_i_*.

**Figure 5:**
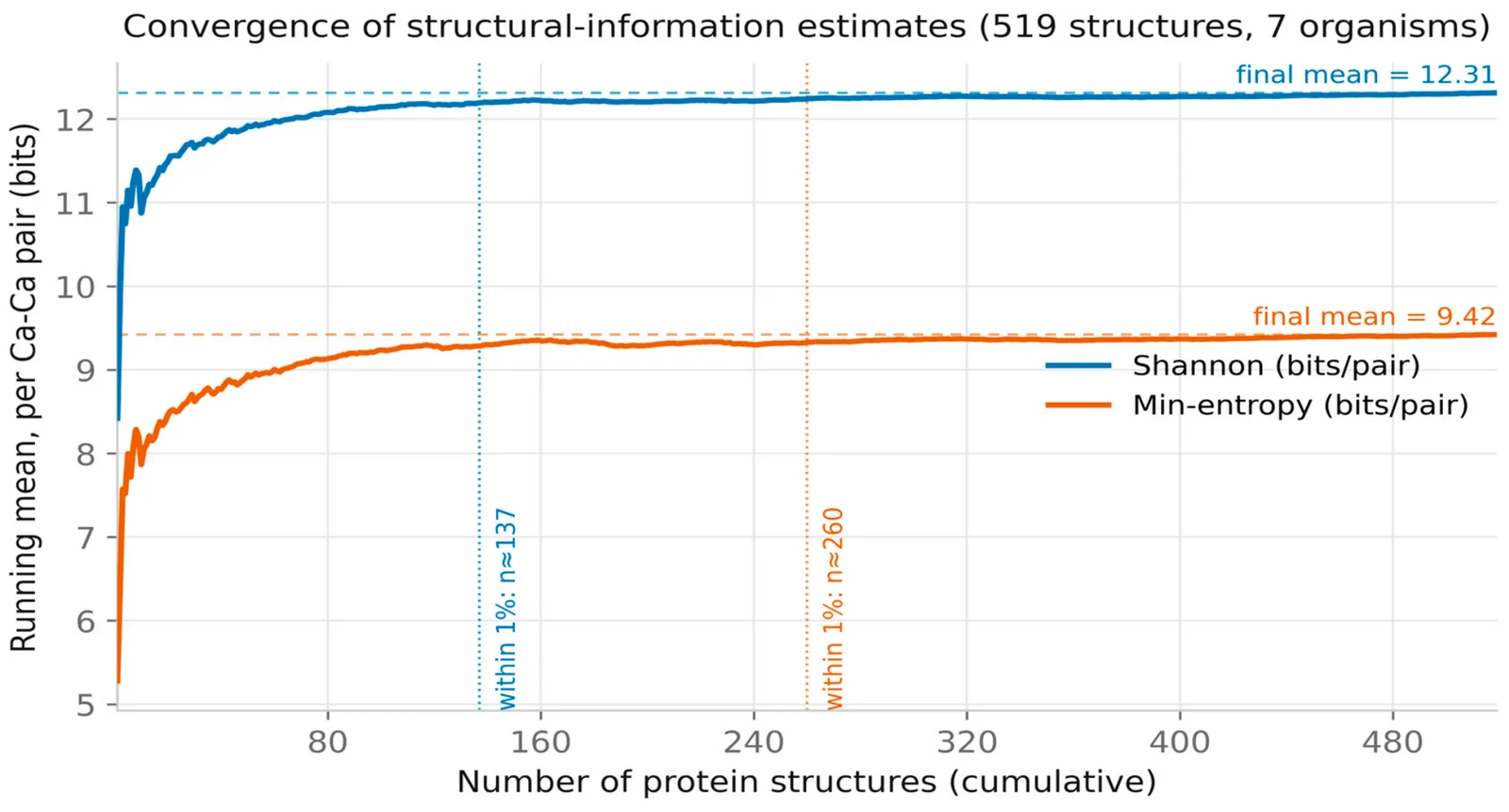
Convergence of the per-residue-pair structural-information estimates (running means of Shannon and min-entropy) as the number of evaluated structures grows to 519 across seven organisms; both stabilize to 12.31 and 9.42 bits, respectively. These are average per-residue-pair descriptive entropies of the within-structure distance distribution, not key-length bits; fractional values are expected for an average over pairs.

*Structural Information Content:* For one structure, let the quantized distances take values indexed by *v* with empirical frequencies *p_iv_* over its m2 residue pairs. We report the per-pair Shannon and min-entropy of that distribution:

(18) $$H_{i}=-\sum_{v}p_{\mathrm{iv}}\log_{2}p_{\mathrm{iv}},\;\;H_{\infty ,i}=-\log_{2}\max_{v}p_{\mathrm{iv}},$$

and average *H_i_* and *H*_∞,_*_i_* across the 519 structures (an unweighted mean over structures, so proteins of different lengths contribute equally). The mean Shannon entropy is 12.31 bits per residue pair (range 6.76 to 14.30) and the mean min-entropy is 9.42 bits per residue pair; Figure 5 shows that the running means change only modestly after the indicated sample sizes under the fixed ordering used in this dataset, indicating substantial empirical diversity in the quantized distance distributions across the evaluated descriptors. These are descriptive empirical entropies of the within-structure distance distribution; residue-pair distances are geometrically dependent, so the reported values are not multiplied by the number of pairs and are not interpreted as cryptographic key entropy.

*Collision-Freedom of the Geometry:* Distinctness of the geometry is checked on the descriptor itself, using an unkeyed SHA-256 of *D_i_* (not the protocol’s seeded credential *k*_bio,_*_i_*, whose distinctness would follow trivially from distinct seeds and would say nothing about geometry). Across the 519 structures, all unkeyed descriptor hashes are distinct, so no two evaluated proteins share the same quantized geometry; we report this as an observation on the sample, not a universal guarantee.

*Selection Entropy and Why the Seed is Needed:* Structural richness is not the security parameter, and we separate the two. An attacker does not guess a random distance matrix but selects among known proteins, so the security-relevant quantity is the size of that selection space. Taking the AlphaFold Protein Structure Database’s coverage of about 2.14 × 10^8^ sequences [15] as an upper bound, the selection space is at most about 2^27.7^, and a real user’s choice is likely far more predictable than uniform over that set; either way it is weak against offline guessing and is an upper bound on selection entropy, not a claim of true user-choice entropy.

This is exactly why the design keys the credential with a secret 128-bit seed in the credential construction and why the security proof rests on Assumption 5 rather than on the protein’s unpredictability: the geometry supplies distinct identity across the sample, while the certified public key and the secret seed anchor identity and supply the brute-force security.

*Reproducibility Check:* All 519 descriptors were regenerated end-to-end with the reference verification tool (SHA-256 ede3c65f91a984be27bfbe8780f9cd4efa3f7442587e137652bf6380b3d9334a; the run environment and per-file digests are recorded in the run manifest). For every structure, the tool records the resolved mmCIF and PDB URLs, the model version, and the mmCIF file SHA-256; in the reported run, all 519 structures resolved to a single uniform model version with residue counts preserved. The check uses two independent references. First, a faithful replay of the original tabulation method (PDB coordinates with two-decimal distance rounding) reproduced the original tabulated statistics for all 519 structures, providing no evidence of a model-version change affecting the reported statistics and confirming that the original method is reproduced exactly.

Second, the normative integer/mmCIF recomputation matches the adopted dataset of record. The two references differ, at the level of the rounded per-structure statistics, in exactly nine rows—only in the fourth decimal of the entropy estimates and by, at most, one in the count of distinct quantized values, with identical residue counts. The tool verifies that every place where the exact-integer and the floating-point quantizers assign different bins falls on an exact half-unit rounding boundary *s* = (10*q* ± 5)^2^, with no non-boundary difference anywhere.

Because the faithful replay reproduces the original statistics (first reference, providing no evidence of a model-version change) and every quantizer difference is a half-unit tie (boundary check), the nine differences are pure round-half-to-even tie-breaking rather than an artefact of the computation. All 519 unkeyed descriptor hashes are distinct (no collisions), the mean Shannon and min-entropy are 12.31 and 9.42 bits per residue pair, and the Shannon range is 6.76 to 14.30; the normative output is adopted as the dataset of record, and no reported aggregate changed.

### 6.3. Symbolic and Executable Security Evidence

Two further experiments corroborate the preceding security analysis. Symbolically, the ProVerif results of Section 5.3 (all six queries holding for *n* = 3 and *n* = 4, and Round 0 shown necessary for injective agreement) corroborate the message logic. Executably, we ran a member-tampering negative control. In each trial, an insider (member 0) replaces the Round 1 contribution of a victim (member 1) with an attacker-chosen point and re-authenticates it, and an honest verifier applies the acceptance rule of the respective design; the 200 trials at each size differ only in fresh randomness (seeds, ephemeral scalars, nonces).

Under a shared-credential Burmester–Desmedt baseline, where one symmetric key authenticates every broadcast, the insider can recompute the authenticator, so the forged contribution is accepted in all 200 of 200 trials at each of *n* = 3,4,5,8. Under PDKE-G the acceptance rule requires a valid ECDSA signature under the victim’s certified public key on a message bound to the victim’s index, the roster, and the session; the insider does not hold the victim’s key, so the forgery is rejected in all 200 of 200 trials.

This is a simplified negative control illustrating the honest-member insertion/substitution class addressed by Corollary 2, not a reproduction of the two-group Sun–Tian experiment; it isolates the single capability the corollary rules out—accepting a party as a participant without its own authenticated contribution. The security cause is precisely the combination the corollary identifies: an independent, PKI-certified signing key per member, the binding of each message to its sender, roster, and context, and verification before acceptance. The protein geometry is the method by which each member’s key is derived; it is not, by itself, the reason the attack fails.

### 6.4. Performance and Scalability

*What Was Measured, and How:* We measured the *per-member* computation time as a function of the group size. For a fixed member, we time the exact operations that member performs in one full protocol run: four ECDSA signatures for Rounds 0 to 2 and the confirmation, the 4(*n* − 1) peer signature verifications, one confirmation-tag computation and *n* − 1 tag checks, the Burmester–Desmedt combination, and the HKDF derivation. Timing uses Python’s perf_counter, reporting the median of nine repetitions after a warm-up run to smooth scheduling noise.

The nine per-repetition timings for each group size, from which the spread of the median can be assessed, are provided in the reproducibility package. The column is not an independent measurement: it is the estimated aggregate computation, $T_{\mathrm{group}}=n\cdot {\tilde{T}}_{\mathrm{member}}$, obtained by multiplying the median per-member time by the group size, and is reported only to make the aggregate cost explicit. The experimental environment is a standard Google Colab CPU runtime: an Intel Xeon CPU at 2.20 GHz (2 vCPUs), about 13 GB of RAM, Linux kernel 6.6, and CPython 3.13.15, with the reference implementation using only the Python standard library; these specifics are captured by an environment cell in the evaluation notebook and reproduced in the package.

Because timings are single-threaded pure-Python arithmetic, absolute values vary with the Colab instance assigned, but the linear per-member trend is stable across runs.

**Figure 6:**
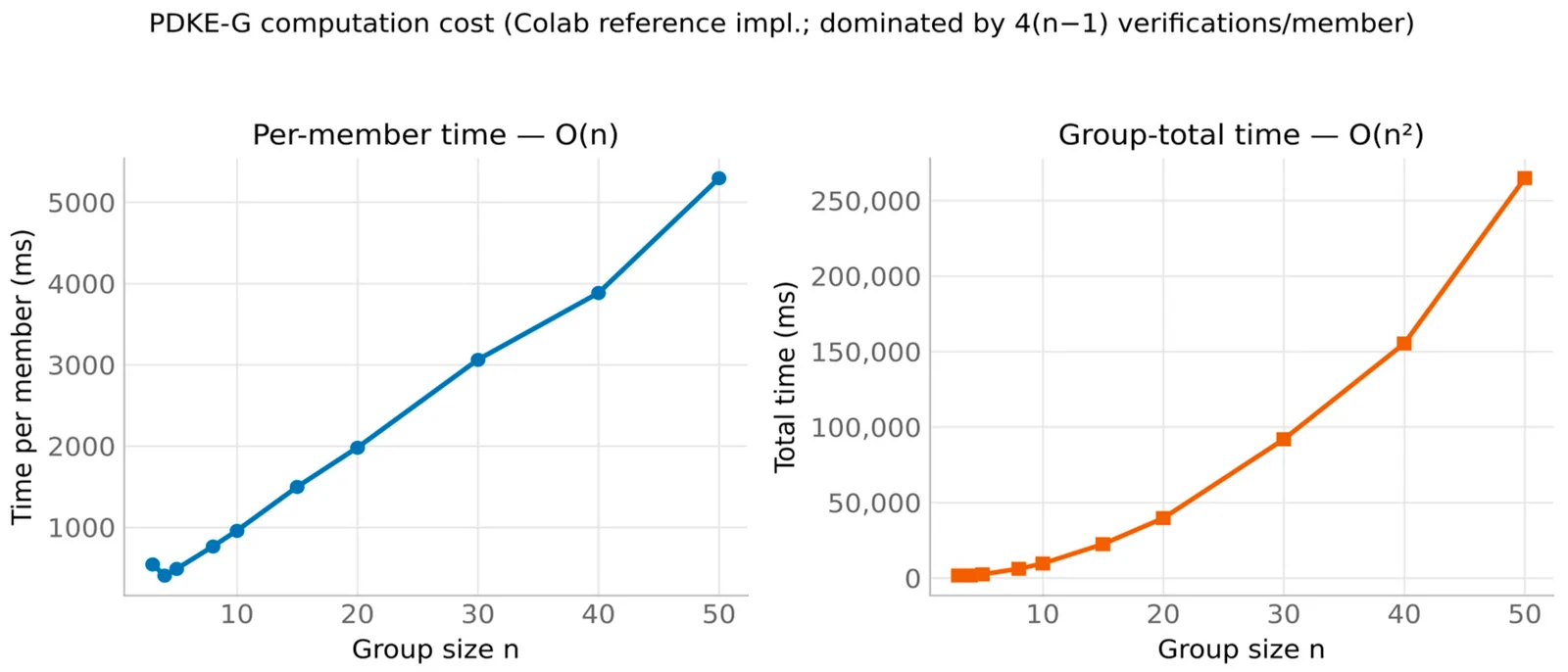
Computation cost: measured per-member time is linear in *n* (**left**); the estimated aggregate *n*⋅ per-member time is quadratic (**right**), matching the 𝒪(*n*) and 𝒪(*n*^2^) analysis.

*Results:* Table 2 collects the numbers. The number of rounds is constant at four for every group size, as specified. The measured per-member time grows approximately linearly with *n*, consistent with the 𝒪(*n*) analysis of Section 4.6 dominated by the 4(*n* − 1) signature verifications (Figure 6, left); an ordinary-least-squares fit over the ten group sizes gives ${\tilde{T}}_{\mathrm{member}}\approx 101.9\;n+3.3$ ms with *R*^2^≈0.994. At the smallest sizes the per-member time is not strictly monotone in *n* (for instance *n* = 4 is measured slightly faster than *n* = 3). This small-size non-monotonicity is consistent with timing variability in the single-threaded pure-Python runtime on the shared Colab instance and does not alter the strong overall linear trend.

**Figure 7:**
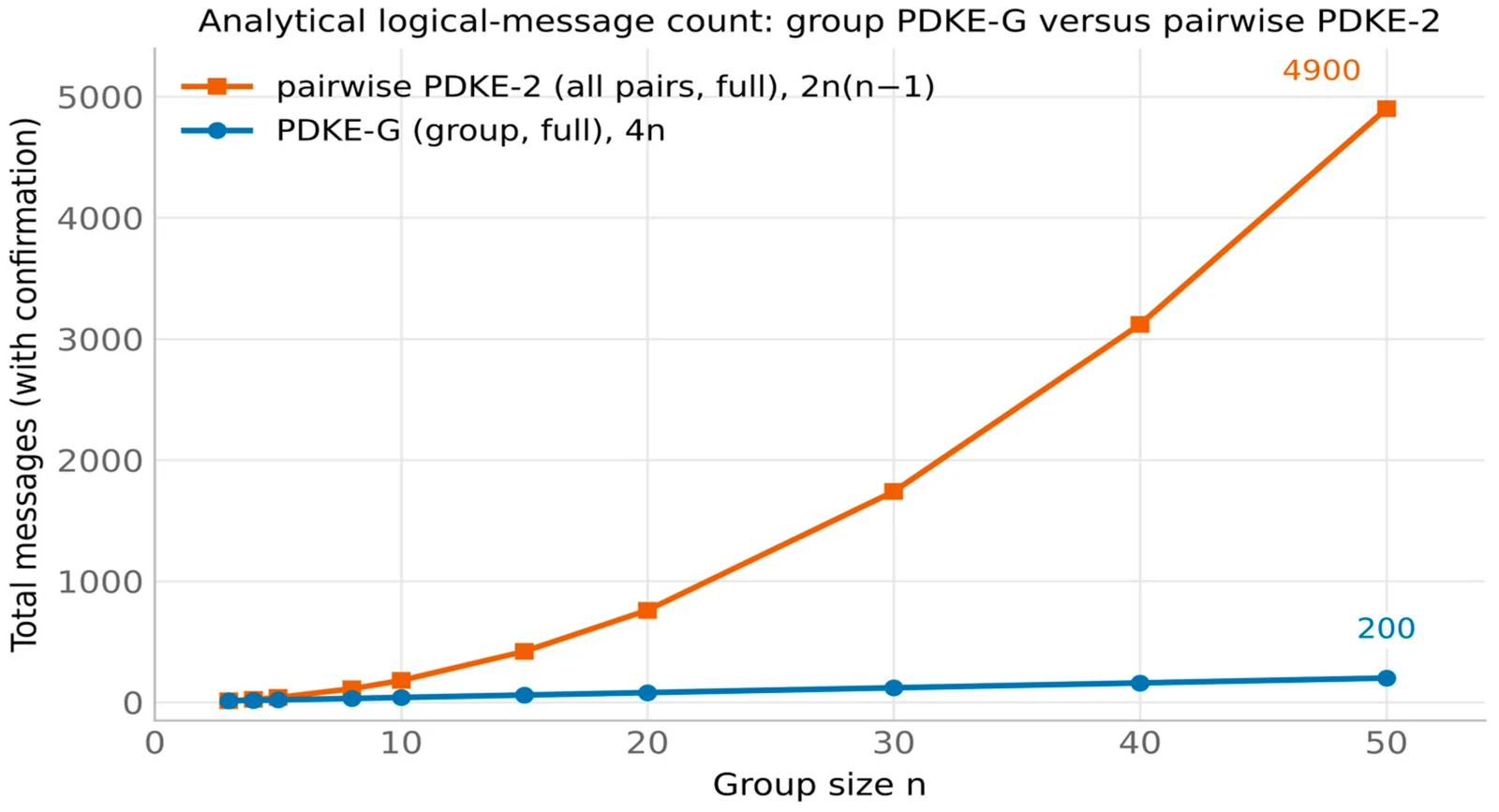
Analytical logical-message count (not a measured network trace): PDKE-G uses 4*n* messages against the 2*n*(*n* − 1) of running the two-party protocol over all pairs.

The group-total column consequently grows as 𝒪(*n*^2^) by construction, since it is *n* times a quantity linear in *n* (Figure 6, right); this is a corollary of the per-member measurement and the analysis, not an independent empirical confirmation of quadratic growth. At the logical-message level the complete protocol uses 4*n* messages against the 2*n*(*n* − 1) of the all-pairs baseline (Figure 7 plots these analytical counts, not a measured network trace); as analyzed in Section 4.6, the advantages are the constant-round count and the single contributory group key, not fewer raw transmissions.

## 7. Discussion

In PDKE-G the protein geometry is a per-member input to credential derivation, while the cryptographic strength comes from the secret seed and standard primitives. The theoretical analysis and experimental evaluation establish key indistinguishability, injective authentication, and, within the scope of Corollary 1, resistance to the honest-member insertion that the negative control illustrates. Because several of these properties are easy to overstate, we state plainly below what the design does and does not provide.

*What the Protein Binding Is, and Is Not:* The protein is a member-specific local input to credential derivation, not a publicly verifiable biometric binding. A verifier cannot check from the certified public key *Q_i_* that any particular protein was used, since *Q_i_* = *d_i_G* reveals nothing about the geometry behind *d_i_*; establishing a verifiable link from a registered participant to a specific structure would require a separately specified enrollment and attestation procedure, which we do not claim. Consequently, PDKE-G is not a biometric or password-authenticated key exchange. The empirical study measures only descriptive structural diversity across a sample of 519 structures and reports no collisions *in* that sample; it is not a universal collision-freedom guarantee, and the reported entropies are within-structure descriptive quantities, not cryptographic key entropy.

*Why the Secret Seed carries the Security:* Because the selection space of known proteins is only about 2^27.7^, and a real user’s choice is likely far more predictable than uniform over that set, the protein alone is weak against offline guessing. Resistance to offline recovery of the long-term credential therefore rests on the secret 128-bit seed *r_i_*, which keys the HMAC extraction so that offline seed recovery is infeasible; identity is further anchored by the certified public key in the PKI.

The practical cost is that each member must generate and store a high-entropy secret seed as securely as any long-term signing key: a deployment that stores it carelessly forfeits the guarantee, and losing it means re-enrollment.

*Scope of the Security Guarantees:* The authentication result (Theorem 2) is an injective agreement restricted to honest peers: for an honest accepting session, every honest peer it believes participated has a unique matching running session. We do not claim mutual acceptance over a fully adversarial network, since the adversary may withhold a confirmation from one party, nor do we claim contributiveness or key-control resistance against arbitrary insider coalitions; these are separate properties outside the present scope. Forward secrecy is post-accept long-term-key forward secrecy and is well defined only under the secure-erasure precondition of Section 4.4: an implementation that fails to erase the ephemeral scalar *a_i_* voids the claim.

The present computational proof models both hash_to_curve and the SHA-256 session-binding map as random oracles. The SHA-256 component alone may instead be expressed through collision resistance plus an explicit unpredictability advantage. This substitution does not remove the random-oracle programming used for hash_to_curve; obtaining a fully standard-model proof would require a different reduction or construction.

*Symbolic Versus Computational Evidence:* The ProVerif models cover only small, fixed group sizes and abstract the Burmester–Desmedt ring through its Diffie–Hellman core, whereas the secrecy of the *n*-party ring key for general *n* is established computationally in Section 5.4. The two forms of evidence are complementary, and neither substitutes for the other; an unbounded-*n* symbolic treatment is left as future work.

*Performance and Deployment:* The reference implementation is single-threaded pure Python intended for reproducibility, not production; per-member work is *O*(*n*), dominated by the 4(*n* − 1) signature verifications, and the efficiency advantages are the constant round count and the single contributory group key rather than a smaller raw transmission count, which remains *Θ*(*n*^2^) over point-to-point links. The potential deployment settings are those that need one shared key among many parties with modest churn—device fleets, sensor groups, and collaborative sessions—over a PKI that certifies each *Q_i_*; production validation and evaluation on real hardware and networks remain necessary before such deployment.

Two operational requirements matter here: the AES-256-GCM data phase requires strict per-sender nonce uniqueness with mandatory key renewal before any counter wraps, and a sender that loses its counter state must trigger a fresh session. The present protocol also assumes a fixed roster per session; dynamic join and leave are handled by re-running the protocol on the new roster.

*Future Work:* Four directions follow directly. First, a post-quantum transition should protect both key establishment and authentication. A hybrid design could combine the present BD secret with an ML-KEM [18] shared secret through a domain-separated combiner to preserve confidentiality unless both components fail, drawing on recent tightly secure lattice-based authenticated key exchange [30]; however, full post-quantum authentication would additionally require replacing or hybridizing ECDSA/P-256 with a standardized post-quantum signature. Merely adding ML-KEM while retaining ECDSA would not provide end-to-end post-quantum security.

Second, an unbounded-*n* symbolic analysis in Tamarin [17] would close the gap between the fixed-size ProVerif models and the general computational proof. Third, robustness across model releases could be studied through a formally specified error-tolerant descriptor or secure-sketch/fuzzy-extractor construction [31]. Such a design would first require an empirical noise model, a distance metric and acceptance threshold, and an analysis of helper-data leakage; the present results do not establish the entropy assumptions needed by a fuzzy extractor. Fourth, the certificate-level structure binding specified in Section 4.2 and Section 5.6 assumes the certifier recomputes the canonical descriptor at enrollment; distributing that check, for example, through a transparency log of structure-of-record commitments or threshold certification, together with structure revocation, are natural extensions.

Efficient support for dynamic membership without a full re-run is a further practical target.

### Deployment Recommendations

We summarize practical measures that minimize risk in a deployment. The per-member secret seed should be generated from a high-entropy source, stored in protected hardware, and never reused across identities. Enrollment should bind each certified public key to its participant through a strict PKI procedure, and every session roster should be checked to contain exactly n distinct registered keys. Where the application must attribute post-agreement traffic to a specific sender, per-sender signatures or sender-specific keys should be used in addition to the shared-key authenticated encryption. The AES-GCM counter state must be managed so that a key is retired before any counter wraps, and a fresh session must be run before a sender that lost its counter state transmits again. Ephemeral scalars and Diffie–Hellman state must be securely erased after a session accepts or aborts, which is required for forward secrecy. Finally, a post-quantum migration path is available by combining the exchange with a standardized lattice key-encapsulation mechanism in a hybrid, which we leave as future work.

## 8. Conclusions

We presented PDKE-G, a contributory group authenticated key exchange whose per-member signing credentials are derived from AlphaFold2-predicted protein geometry as a member-specific input. Each member derives its long-term signing key by HKDF extraction from the quantized Cα–Cα distance matrix of its own protein under a normative, byte-reproducible canonicalization profile, keyed by a secret 128-bit seed. A constant-round Burmester–Desmedt core, authenticated by roster- and session-bound per-member signatures with nonce precommitment and a signed confirmation round, yields a single 256-bit session key with forward secrecy in four broadcast rounds.

We gave a computational proof for general group size, reducing key indistinguishability to DDH (with the passive Burmester–Desmedt advantage invoked as a published black box), the KDF security of HKDF, credential-derivation pseudorandomness, and SUF-CMA of the instantiated signature, in the random-oracle model for the hash-to-curve and SHA-256 maps. We proved injective agreement for honest peers under insider corruption and, as Corollary 2, resistance to honest-member insertion. The three- and four-party instantiations were verified symbolically in ProVerif, and an executable negative control illustrated the insertion capability the corollary rules out.

The biology’s role is narrowly bounded: the protein geometry is a member-specific local input, not a secret, though its binding to the member’s structure of record is made publicly verifiable by certificate attestation (Section 4.2 and Section 5.6), so resistance to offline credential recovery rests on the secret seed and identity on the certified public key, while session-key confidentiality rests on the ephemeral group secret, DDH, and the stated KDF assumptions rather than on the protein’s unpredictability. The security theorem is conditional on its stated assumptions, and the reference implementation is a single-threaded reproducibility artefact, not a production system. Of the future directions set out in the Discussion, two stand out: a post-quantum transition combining the elliptic-curve core with a standardized lattice key-encapsulation mechanism [18] and a post-quantum signature, and an unbounded symbolic analysis in Tamarin [17]. Overall, PDKE-G shows that per-member biological geometry can serve as a reproducible member-specific input in a group protocol when its role is scoped conservatively and its security is carried by standard, well-understood primitives.

## Figures and Tables

**Table 1:** Correspondence between PDKE-G elements and the published primitives/protocol components.

PDKE-G Element	Published Counterpart
z_i = a_i·G*	Round-1 Burmester–Desmedt contribution
X_i = a_i(z_{i + 1} − z_{i −1})	Round-2 Burmester–Desmedt ring value
K = j ajaj + 1 G*	Burmester–Desmedt group secret
sid = ctx	Katz–Yung compiler session identifier
Round 0 to Round 2 signatures	Katz–Yung authenticated compiler flows
confirmation tagi,σiconf	additional key-confirmation layer
Reveal, Corrupt, Test	corresponding Katz–Yung/Bresson–Chevassut–Pointcheval experiment queries

**Table 2:** Rounds, measured median per-member time, estimated aggregate group time (*n*⋅ per-member), and analytical message counts versus group size *n* (Google Colab reference implementation). The group-total column is *n* times the full-precision median per-member time, computed before the per-member column was rounded for display, so it may differ in the last digit from *n* times the rounded value shown.

n	Rounds	Per-Member (ms)	Group-Total (ms)	Core Msgs (3n)	Full Msgs (4n)	Pairwise (2n(n − 1))
3	4	545.30	1635.9	9	12	12
4	4	405.26	1621.1	12	16	24
5	4	488.08	2440.4	15	20	40
8	4	763.80	6110.4	24	32	112
10	4	958.04	9580.4	30	40	180
15	4	1496.97	22,454.6	45	60	420
20	4	1981.09	39,621.8	60	80	760
30	4	3063.12	91,893.5	90	120	1740
40	4	3883.72	155,348.7	120	160	3120
50	4	5295.98	264,798.7	150	200	4900

## Data Availability

The reference implementation, ProVerif models, entropy dataset (PDKE-G_entropy_normative_record.csv, with the unmodified original PDKE-G_entropy_original_v4.csv), the descriptor, credential-derivation, and three-party transcript known-answer test vectors, the ablation study, canonicalization verification tool and its run manifest, and evaluation notebooks will be made available as a reproducibility package with the published article.

## Supplementary Materials

The following supporting information can be downloaded at: https://www.mdpi.com/article/10.3390/biomimetics11100753/s1, Script S1: PDKE-G_binding_integrity_demo.py (binding-integrity control); Text S1: PDKE-G_binding_integrity_output.txt (output of Script S1).
